# Expediated synthesis of *N*-acyl-*N*-alkyl sulfonamide probes for protein proximity labelling

**DOI:** 10.1039/d6ob00339g

**Published:** 2026-03-26

**Authors:** Phathutshedzo Masithi, Laetitia Raynal, Peter O'Brien, Christopher D. Spicer

**Affiliations:** a Department of Chemistry and York Biomedical Research Institute, University of York Heslington YO10 5DD UK chris.spicer@york.ac.uk

## Abstract

*N*-Acyl-*N*-alkysulfonamides (NASAs) can be used to site-selectively label proteins *via* proximity-mediated reactions. However, the synthesis of NASA probes can be challenging, typically using a two-step synthetic route that relies on alkylation of a weakly nucleophilic acyl-sulfonamide intermediate. Here, we develop a novel one-step strategy for NASA synthesis from a common alkyl-sulfonamide intermediate, overcoming these challenges. A series of NASA probes bearing common labels for protein modification are efficiently synthesized, in a single step, from the corresponding carboxylic acid. This method will increase the accessibility and versatility of these powerful reagents, and the applications of proximity-mediated protein labelling.

## Abstract 2

A streamlined synthetic route to access *N*-acyl-*N*-sulfonamide (NASA) probes for protein labelling has been developed.

## Graphical abstract

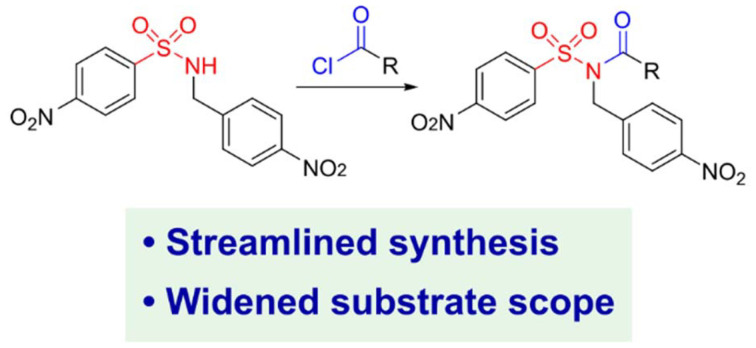

Proximity-mediated labelling is at the forefront of efforts to site-selectively modify proteins.^1^ In these approaches, ligand binding is used to bring a reactive group into close proximity with specific amino acids on the protein surface, inducing labelling in a regio-selective manner.^2^ Importantly, this reactive group must possess insufficient reactivity to label the protein when free in solution. Ligand binding creates a pseudo-intramolecular environment with high effective molarity, enabling labelling to take place.^3^

Among the probes developed for proximity-mediated labelling, *N*-acyl-*N*-alkyl-sulfonamides (NASAs, 1), first identified by the Hamachi group, have emerged as leading candidates.^4–6^ These electrophilic acylating reagents react preferentially with lysine residues and N-terminal amines only when brought into close contact with the protein, with tunable reactivity dependent on the nature of sulfonamide substitution.

An extension of NASA chemistry lies in catalyst-mediated proximity labelling.^7^ In this approach, the protein-binding ligand is modified with a pyridinium oxime nucleophilic catalyst, 2, which activates the NASA to form a moderately reactive *O*-acyl oxime intermediate (Scheme 1a). In a ligand–protein complex (3), this intermediate will acylate nearby nucleophilic residues, regenerating the pyridinium oxime catalyst. In contrast, when free in solution the *O*-acyl oxime intermediate has insufficient reactivity to label the protein, and ultimately undergoes hydrolysis. As a result, labelling efficiencies correlate strongly with the protein binding affinity of the ligand.^4^

**Scheme 1:**
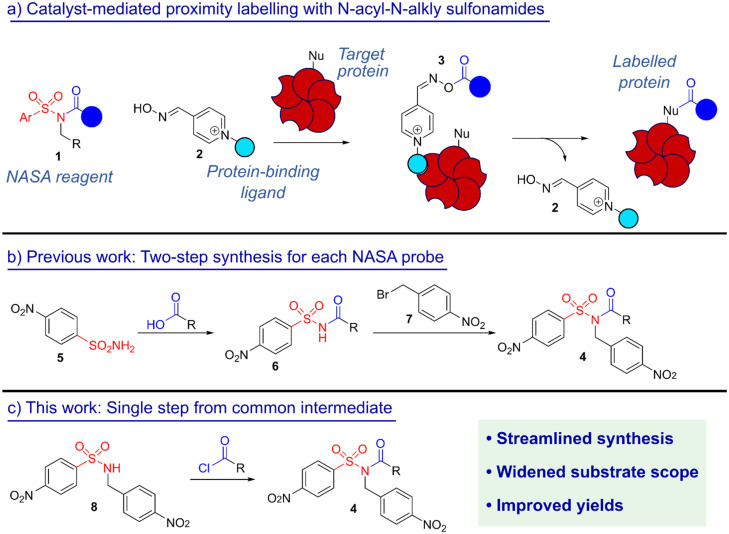
(a) Schematic of catalyst-mediated proximity labelling; (b) previously reported two-step route to the synthesis of NASA reagents; and (c) the simplified one-step synthesis reported here from the common sulfonamide intermediate 5.

Catalyst-mediated proximity labelling is attractive as it decouples the protein-binding ligand and the label of interest: a single catalyst-modified ligand can be used to modify proteins with a wide range of NASAs bearing different labels of interest, streamlining and simplifying reagent synthesis.

*N*-(4-Nitrobenzyl)-*N*-(4-nitrophenyl) acylsulfonamides (referred to here as NO_2_Bz-NASAs, 4) are one class of NASA that have been reported as suitable reagents for this catalyst-mediated labelling approach. They provide an ideal balance between sufficient reactivity to react efficiently with pyridinium oxime catalysts, while exhibiting minimal off-target/non-proximity induced reactivity.^7^

In recent work, we synthesised a series of NO_2_Bz-NASAs during our development of peptide-based pyridinium oxime catalysts for protein labelling.^8^ The traditional approach to synthesise these NASA probes is to first acylate 4-nitrobenzenesulfonamide 5 with the required label, followed by subsequent alkylation of the resultant *N*-acylsulfonamide 6 with 4-nitrobenzyl bromide 7, to generate NASAs of the form 4 (Scheme 1b). While this approach was suitable for the synthesis of some of our target probes, we encountered a number of challenges: (i) the alkylation of 6 was often challenging and sluggish, due to the weak nucleophilicity of the *N*-acylsulfonamide nitrogen. This resulted in low yields for some targets, and unsuccessful syntheses in other cases; (ii) if the probe contained an amide, benzylation was favoured at this site rather than the desired, but less nucleophilic, acylsulfonamide. This in turn limited the scope of probes that could be generated; and (iii) synthesis of each NO_2_Bz-NASA required two synthetic steps, with purification by flash column chromatography after both. When probes were only available in low quantities, or were highly hydrophilic, these two purification steps became problematic.

To overcome these challenges, we here report a single-step approach to NASA-synthesis from common, pre-formed *N*-alkyl-sulfonamide intermediates (Scheme 1c). This approach is applicable to the synthesis of a diverse range of probes and NASA-substituent patterns, and will enhance the widespread adoption of this powerful class of reagent in protein labelling.

## Results and discussion

We envisaged that the synthesis of *N*-(4-nitrobenzyl)-*N*-(4-nitrophenyl) sulfonamide 8 would provide a common reagent for the single-step, divergent synthesis of a range of NO_2_Bz-NASA probes. 8 was initially generated in a 76% yield through the reaction of sulfonyl chloride 9 and 4-nitrobenzylamine 10 at room temperature in DCM, with 3 equiv. of DIPEA as a base (Table 1, entry 1). However, during this reaction significant quantities of side-products resulting from diacylation were generated. This led to poor reproducibility and diminished yields at higher scale. Side product formation could be eliminated by first adding 10 at −78 °C, with the reaction then being allowed to warm to room temperature. However, this improvement came at the expense of overall conversion (entry 2). Changing the solvent to THF and the base to triethylamine, and gradually heating the reaction to reflux after initially adding 10 at −78 °C improved yields to 82% (entry 3). Further optimisation, reducing the number of equivalents of base to 1.5 equiv., eventually led to an improved yield of 96% on a multi-gram scale.

**Table 1:** Optimisation of the synthesis of *N*-(4-nitrobenzyl)-*N*-(4-nitrophenyl) sulfonamide 5

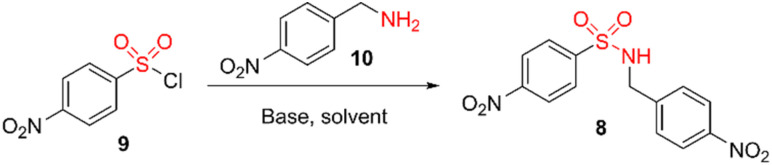				
Entry	Base (eq.)	Solvent	Temp. (°C)	Yield (%)
1	DIPEA (3.0)	DCM	rt	76
2	DIPEA (3.0)	DCM	−78 then rt	31
3	NEt_3_ (3.0)	THF	−78 then 66	82
4	NEt_3_ (1.5)	THF	−78 then 66	96

With intermediate 8 in hand, we looked to generate hexyl-NO_2_Bz-NASA 11 from hexanoic acid as a simple model substrate. Direct amide coupling of 8 with hexanoic acid, under analogous conditions to those used traditionally to synthesize *N*-acylsulfonamides (6) during a two-step NASA synthesis, proved unsuccessful. The reaction of 8 with hexanoic acid *N*-hydroxysuccinimide ester similarly failed to generate the desired NASA probe 11.

**Scheme 2:**
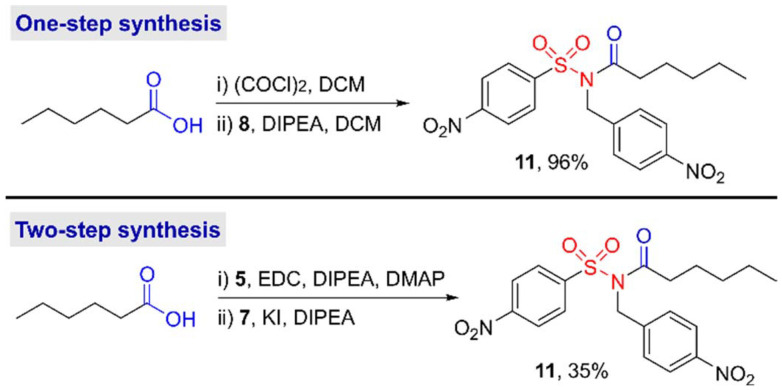
Comparison of the synthesis of hexanoic-NASA 8*via* either the one-step synthesis reported here, or a previously reported two-step strategy.

These results are in contrast to the work of Kawano *et al.* who previously reported an analogous strategy for the synthesis of *N*-cyanomethyl- and methyl-sulfonamides.^9^ In their work, couplings with EDC and catalytic DMAP were found to deliver the desired acylated reagents. However, they also demonstrated that the probes could be generated from the corresponding acyl fluoride, generated *in situ* with bis(tetramethylene)fluoroformamidinium hexafluorophosphate (BTFFH). Similarly, here we found that when 8 was reacted with hexanoyl chloride, generated from the treatment of hexanoic acid with oxalyl chloride immediately prior to use, the desired NO_2_Bz-NASA 11 was generated in a 96% yield (Scheme 2). This high single step yield compares favourably with the overall 35% yield obtained in our previous report, when 11 was synthesised *via* the traditional two-step synthetic route using acyl sulfonamide 6.^8^

**Scheme 3:**
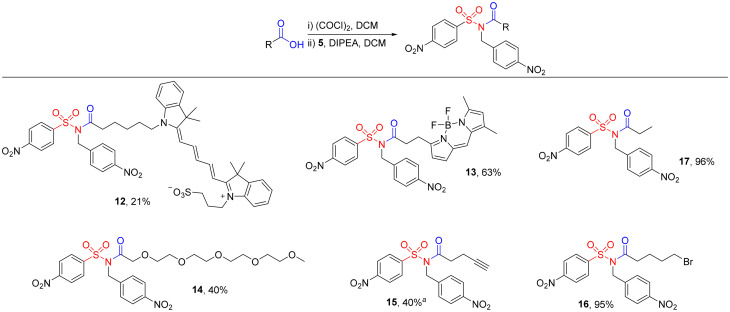
Synthesis of NO_2_Bz-NASAs. ^*a*^NASA 15 was synthesised from the corresponding carboxylic acid using Mukaiyama reagent. Conditions: 5 (1.0 equiv.), carboxylic acid (1.5 equiv.), Mukaiyama reagent (1.5 equiv.), NEt_3_ (3.0 equiv.), THF, rt, 17 h.

We went on to demonstrate the benefits of 8 as a common intermediate for NO_2_Bz-NASA synthesis (Scheme 3). NASA probes for protein functionalisation, including fluorophores (12 and 13) and oligo(ethylene glycol) chains (14) could all be produced efficiently in a single step. The synthesis of Cy5 dye 12 is particularly noteworthy. We had synthesized this probe in our previous report *via* a two-step approach, but purification at each stage was challenging due to the hydrophilic, zwitterionic nature of both the desired NASA and intermediate acyl sulfonamide, with limited separation on normal phase silica.^8^ By performing only a single operation, on this substrate, 12 could be obtained more simply here in a 21% yield.

Importantly, our approach also allowed the synthesis of probes containing handles for further derivatisation, including alkyne 15 for copper-catalysed azide–alkyne cycloaddition, and alkyl bromide 16 for alkylation. In the case of 15, we found that NASAs could be accessed directly from the corresponding carboxylic acid using Mukaiyama reagent, despite attempted couplings with other amide coupling reagents such as EDC having been unsuccessful, as described above. Notably, the low nucleophilicity of the sulfonamide of 8, and therefore the requirement to use highly activated carboxylic acids limits compatibility of this method with substrates bearing other unprotected nucleophiles, such as amines, alcohols, and phenols.

**Scheme 4:**
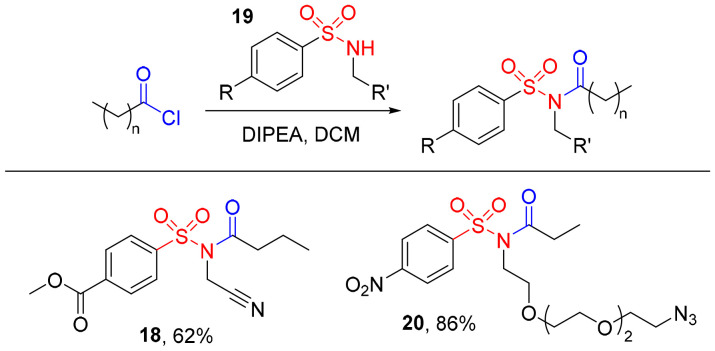
Synthesis of alternative NASA reagents from the corresponding *N*-alkyl-sulfonamide, 19.

Finally, we considered that our approach could find wider utility if it allowed the synthesis of alternative NASA-reagents (Scheme 4). Cyanomethyl-NASAs are more reactive than their NO_2_Bz-analogues, allowing proximity-mediated protein labelling even in the absence of a catalyst.^4^ As such we prepared cyanomethyl-NASA 18 from the corresponding cyanomethylsulfonamide 19a in a 62% yield. Similarly, an oligo-ethylene glycol-NASA 20, bearing an azide at its terminus for further derivatisation *via* copper- or strain-promoted azide–alkyne cycloaddition, was synthesised in an 86% yield from the corresponding sulfonamide 19b. These syntheses highlight the potential for our route to simplify and accelerate the production of a diverse range of NASA probes, including those with poor functional group compatibility with the traditional two-step synthetic route.

## Conclusions

In this paper we have developed a novel one-step strategy for the synthesis of NASA probes for proximity-mediated protein labelling. This strategy makes use of alkyl-sulfonamides as common intermediates, that can be used to modify a wide range of carboxylic acids with relevant functionalities to bioconjugation. Importantly, this strategy overcomes the limitations of the previously used two-step strategy for NASA synthesis, simplifying and accelerating probe development. We therefore anticipate that our method will be of great use to researchers using this powerful strategy for site-selective protein modification, diversifying the labels that are compatible with catalyst-mediated labelling.

## Author contributions

Phathutshedzo Masithi and Laetitia Raynal contributed equally to this manuscript. PM and LR designed and performed all experiments and analysed data. POB and CDS conceptualised the study. CDS supervised the study, and wrote the manuscript. All authors contributed to the review and editing of the manuscript.

## Conflicts of interest

The authors declare no competing financial interests.

## Supplementary Material

OB-024-D6OB00339G-s001

## Data Availability

The data supporting this research is openly available from the research data repository of the University of York at https://doi.org/10.15124/ee255047-f9fc-48b7-8aa8-e1e0971daf31. Supplementary information (SI): all experimental details and NMR spectra of all novel compounds. See DOI: https://doi.org/10.1039/d6ob00339g.

## References

[cit1] Shiraiwa K., Cheng R., Nonaka H., Tamura T., Hamachi I. (2020). Cell Chem. Biol..

[cit2] Hamachi I., Tamura T., Nonaka H. (2025). Acc. Chem. Res..

[cit3] Tamura T., Hamachi I. (2019). J. Am. Chem. Soc..

[cit4] Tamura T., Ueda T., Goto T., Tsukidate T., Shapira Y., Nishikawa Y., Fujisawa A., Hamachi I. (2018). Nat. Commun..

[cit5] Tamura T., Hamachi I. (2025). Acc. Chem. Res..

[cit6] Thimaradka V., Hoon J., Heroven C., Aricescu A. R., Yuzaki M., Tamura T., Hamachi I. (2021). Bioorg. Med. Chem..

[cit7] Tamura T., Song Z., Amaike K., Lee S., Yin S., Kiyonaka S., Hamachi I. (2017). J. Am. Chem. Soc..

[cit8] Raynal L., Nabarro J., Miller L. M., Dowle A. A., Moul S. L., Masithi P., Johnson S. D., Fascione M. A., Spicer C. D. (2025). ACS Omega.

[cit9] Kawano M., Murakawa S., Higashiguchi K., Matsuda K., Tamura T., Hamachi I. (2023). J. Am. Chem. Soc..

