# Supplementary material for: Expediated synthesis of N-acyl-N-alkyl sulfonamide probes for protein proximity labelling
Source: Org Biomol Chem. 2026 Mar 26;24(15):3223–6. doi: 10.1039/d6ob00339g (PMC13054794; doi:10.1039/d6ob00339g)

This journal is © The Royal Society of Chemistry 2026

Expediated synthesis of N-acyl-N-alkyl sulfonamide probes for protein proximity labelling

Phathutshedzo Masithi,[a†] Laetitia Raynal,[a†] Peter O'Brien[a] and Christopher D. Spicer*[a]

[a]Department of Chemistry and York Biomedical Research Institute, University of York, Heslington, YO10 5DD, UK.

Table of contents

S2 General considerations

S3 Sulfonamide synthesis

S6 Substrate synthesis

S10 NASA synthesis

S15 References

S16 NMR spectra of novel compounds

General considerations

Proton and carbon nuclear magnetic resonance (^1H and ^{13}C NMR respectively) spectra were recorded on a Jeol ECX-400 (400 MHz). NMR shifts were assigned using COSY, HSQC and HMBC spectra. All chemical shifts are quoted on the δ scale in ppm using residual solvent as the internal standard (^1H NMR: $CDCl_3$ = 7.26; MeOD = 3.31; DMSO-d_6 = 2.50; Acetone-d_6 = 2.05 and ^{13}C NMR: $CDCl_3$ = 77.16, MeOD = 49.00, DMSO-d_6 = 39.52; Acetone-d_6 = 29.82). Coupling constants (*J*) are reported in Hz with the following splitting abbreviations: s = singlet, d = doublet, t = triplet, q = quartet, m = multiplet, app = apparent, br = broad. Melting points (m.p.) were recorded on a Gallenkamp melting point apparatus. Infrared (IR) spectra were recorded on a Perkin Elmer UATR Two FT-IR spectrometer or a Bruker Alpha II ATR spectrometer with Opus build 8.5.29. High resolution electrospray ionisation (ESI) mass spectra (HRMS) were recorded on a Bruker Compact TOF-MS or a Jeol AccuTOF GCx-plus spectrometer. Nominal and exact *m/z* values are reported in Daltons (Da).

Thin layer chromatography (TLC) was carried out using aluminium backed sheets coated with 60 F_{254} silica gel (Merck). Visualization of the silica plates was achieved using a UV lamp (λ_{max} = 254 nm), potassium permanganate (5% $KMnO_4$ in 1M NaOH with 5% potassium carbonate), or ninhydrin (1.5% ninhydrin, 3% AcOH in *n*-butanol). Flash column chromatography was carried out using Geduran Si 60 (40-63 µm) (Merck). Mobile phases are reported as % volume of more polar solvent in less polar solvent. Anhydrous solvents were dried over a PureSolv MD 7 Solvent Purification System. Deionized water was used for chemical reactions. All other solvents were used as supplied (Analytical or HPLC grade), without prior purification. Reagents were purchased from Sigma-Aldrich, VWR, or Fluorochem and used as supplied, unless otherwise indicated. Brine refers to a saturated solution of sodium chloride. Petrol refers to the fraction of petroleum ether boiling in the range 40-60 °C. Anhydrous magnesium sulfate ($MgSO_4$) was used as the drying agent after reaction workup unless otherwise stated.

1. Sulfonamide synthesis

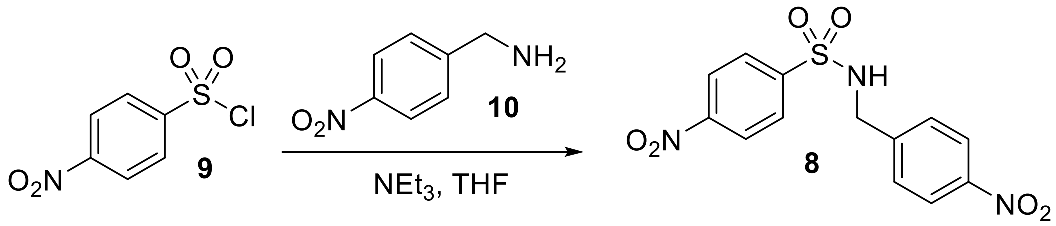

A solution of 4-nitrobenzylamine hydrochloride **10** (6.5 g, 35 mmol) and triethylamine (4.8 mL, 35 mmol) in THF (100 mL) was cooled to -78 °C. A solution of 4-nitrobenzene-1-sulfonyl chloride **9** (5.0 g, 23 mmol) in THF (50 mL) was then added dropwise over 10 min. The reaction mixture was kept at -78 °C for 30 min, before warming up to room temperature and then heating to reflux for 17 h. The reaction was then cooled to room temperature, diluted with hydrochloric acid (1 M, 100 mL), and extracted with EtOAc (3 × 100 mL). The combined organic layers were washed with sat. $NaHCO_3$ (100 mL), dried over $MgSO_4$, filtered and concentrated under vacuum to yield the product as a white solid (6.7 g, 19.8 mmol, 87%)

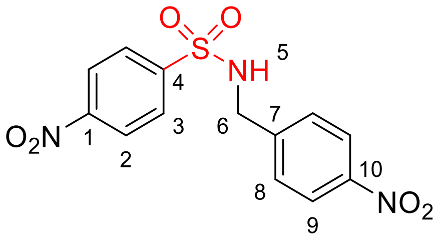

^{1}**H NMR** (400 MHz, DMSO-d_6): δ = 8.77 (1H, t, *J* = 6.3 Hz, H5), 8.37 (2H, d, *J* = 8.7 Hz, H2), 8.13 (2H, d, *J* = 8.7 Hz, H9), 8.02 (2H, d, *J* = 8.7 Hz, H3), 7.50 (2H, d, *J* = 8.7 Hz, H8), 4.23 (2H, d, *J* = 6.3 Hz, H6); ^{13}**C NMR** (101 MHz, DMSO-d_6) δ = 149.6 (C1), 146.7 (C10), 146.2 (C4), 145.4 (C7), 128.7 (C2), 128.1 (C9), 124.6 (C3), 123.5 (C8), 45.4 (C6); **HRMS**: *m/z* (ESI^-) calc. for $C_{13}H_{10}N_3O_6S$ $[M-H]^-$ = 336.0296, obs. = 336.0292; **IR** (film, $υ_{max}$/ cm^{-1}) 3263 (NH), 1508 (N-O), 1343 (S=O); **M.p.**: 170 – 173 °C.

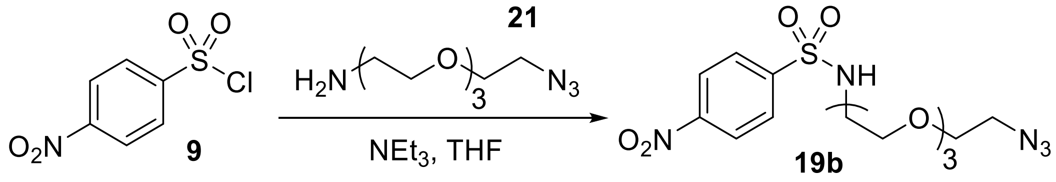

A solution of azide **21** (118 mg, 0.68 mmol) and triethylamine (95 µL, 1.29 mmol) in THF (10 mL) was cooled to -78 °C. A solution of 4-nitrobenzene-1-sulfonyl chloride **9** (100 mg, 0.45 mmol) in THF (5 mL) was then added dropwise over 10 min. The reaction mixture was kept at -78 °C for 30 min, before warming up to room temperature and then heating to reflux for 17 h. The reaction was then cooled to room temperature, diluted with hydrochloric acid (1 M, 10 mL) and extracted with EtOAc (3 × 10mL). The combined organic layers were washed with sat. $NaHCO_3$ (10 mL), dried over $MgSO_4$, filtered and concentrated under vacuum. The residue was purified by column chromatography eluting with 10-30% EtOAc:Petrol. Pure fractions were concentrated under vacuum to afford the product as a yellow solid (120 mg, 0.30 mmol, 67%).

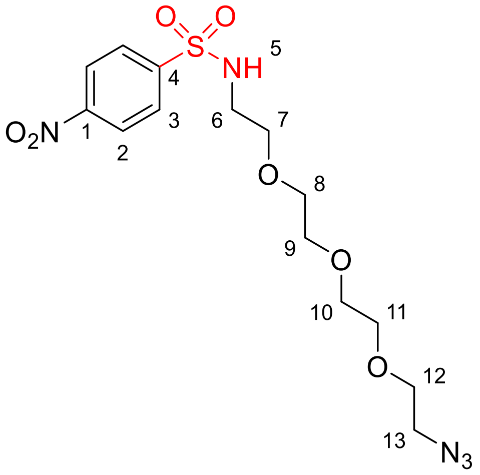

^{1}H NMR (400 MHz, $CDCl_3$) δ = 8.34 (2H, d, *J* = 8.9 Hz, H2), 8.06 (2H, d, *J* = 8.9 Hz, H3), 5.73 (1H, t, *J* = 5.8 Hz, H5), 3.63-3.69 (6H, m, H7, H11, & H12), 3.57-3.60 (2H, m, H10), 3.49-3.55 (4H, m, H8 and H9), 3.36-3.41 (2H, m, H13), 3.16-3.21 (2H, m, H6); **^{13}C NMR** (101 MHz, $CDCl_3$) δ = 150.1 (C1), 146.3 (C4), 128.4 (C2), 124.4 (C3), 70.7 (C12), 70.6 (C11), 70.6 (C7), 70.3 (C10), 70.1 (C9), 69.1 (C8), 50.7 (C13), 43.2 (C6); **HRMS**: *m/z* (ESI$^+$) calc. for $C_{14}H_{21}N_5NaO_7S$ $[M+Na]^+$ = 426.1059; obs. = 426.1051; **IR** (film, υ_{max}/ cm^{-1}) 2873 (NH), 2103 (N_3), 1529 (N-O), 1348 (S=O), 1090 (C-O).

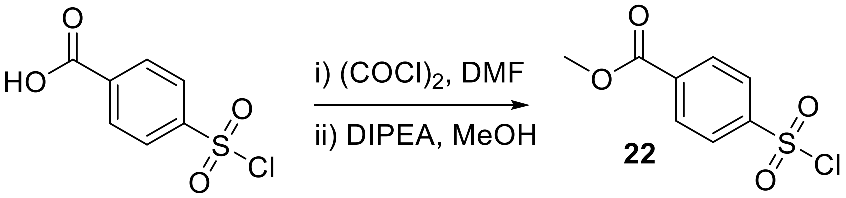

Oxalyl chloride (1.6 mL, 8.4 mmol) was added dropwise to a suspension of 4-chlorosulfonylbenzoic acid (1.1 g, 5.0 mmol) and DMF (50 µL) in anhydrous DCM (15 mL). The reaction was stirred at rt for 2 h and then concentrated under vacuum. The residue was dissolved in DCM (2 mL) and added dropwise to mixture of MeOH (1 mL) and DIPEA (1.3 mL, 13.6 mmol) in DCM (10 mL) at 0 °C, then allowed to warm to rt and stirred for 2 h. The solvents were removed under vacuum, and the residue dissolved in EtOAc (50 mL). The organics were washed with a sat. NH_4Cl (50 mL) and brine (2 × 50 mL), dried over Na_2SO_4, filtered and concentrated under vacuum to afford **22** as a brown solid (1.0 g, 4.3 mmol, 85%). Data were consistent with those previously reported.[21]

^{1}H NMR (400 MHz, $CDCl_3$) δ = 8.27 (2H, d, *J* = 8.9 Hz, 2H, Ar<u>H</u>2), 8.12 (2H, d, *J* = 8.9 Hz, Ar<u>H</u>3), 3.99 (3H, s, -C<u>H</u>$_3$).

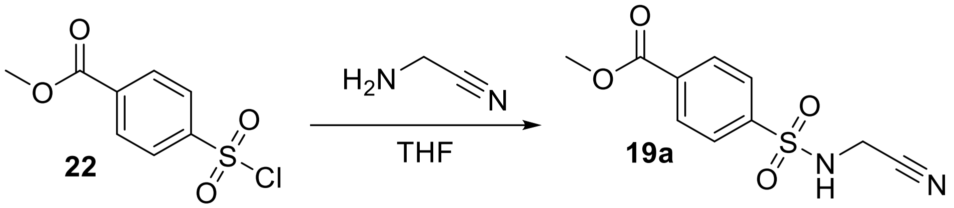

Aminoacetonitrile hydrochloride (67 mg, 0.72 mmol) was added to a solution of sulfonyl chloride **22** (123 mg, 0.48 mmol) and DIPEA (0.25 mL, 1.44 mmol) in THF (10 mL) at 0 °C. After 1.5 h, the reaction mixture was warmed to room temperature and stirred for another 5 h. The reaction was then concentrated under vacuum, and the residue dissolved in EtOAc (20 mL). The organics were washed with H_2O (2 × 10 mL) and with brine (5 mL), dried over Na_2SO_4, filtered, and concentrated under vacuum. The residue was purified by flash column chromatography eluting with 25% EtOAc:Petrol. Pure fractions were concentrated under reduced pressure to afford **19a** as a white solid (45 mg, 0.19 mmol, 39%).

^{1}H NMR (400 MHz, $CDCl_3$) δ = 8.23 (d, *J* = 8.4 Hz, 2H, Ar<u>H</u>2), 7.98 (d, *J* = 8.4 Hz, 2H, Ar<u>H</u>3), 5.14 (s, 1H, -N<u>H</u>), 4.09 (s, 2H, -C<u>H</u>$_2$NH), 3.97 (3H, s, -C<u>H</u>$_3$). **^{13}C NMR** (101 MHz, $CDCl_3$): δ 165.9 (-<u>C</u>O$_2$Me), 143.0 (Ar<u>C</u>1), 135.3 (Ar<u>C</u>4), 131.2 (Ar<u>C</u>2), 127.8 (Ar<u>C</u>3), 114.8 (-CN), 53.2 (-<u>C</u>H$_3$), 31.5 (-<u>C</u>H$_2$NH). **HRMS** (ESI$^-$): *m/z*: calculated for $C_{10}H_9N_2O_4S$

[M-H]$^-$ = 253.0289; obs. = 253.0289; **IR** (film, υ_{max}/ cm^{-1}) 2875 (NH), 1342 (S=O), 1285 (C-O ester); **M.p**: 124 – 126 °C.

2. Substrate synthesis

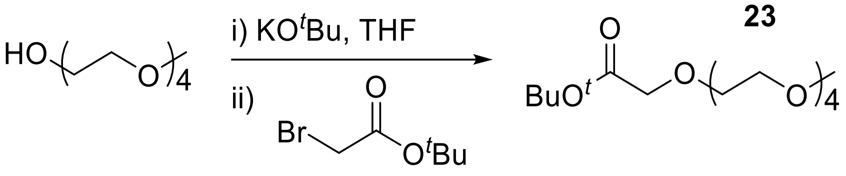

Potassium *tert*-butoxide (1.1 g, 9.8 mmol) was added to a solution of triethylene glycol monomethyl ether (1 mL, 4.6 mmol) in *tert*-butanol (20 mL) and the mixture stirred for 2 h. *Tert*-butyl bromoacetate (2.4 mL, 16.4 mmol) was then added and the mixture stirred for a further 18 h, before being filtered through Celite and concentrated under vacuum to afford the product **23** as a colourless oil (1.4 g, 4.3 mmol, 94%). Data were consistent with those previously reported.[1]

^{1}H NMR (400 MHz, $CDCl_3$) δ = 3.62-3.74 (2H, m, -C<u>H$_2$</u>COOH), 3.25-3.44 (14H, m, PEG), 3.13-3.25 (2H, m, PEG), 3.09 (3H, s, -OC<u>H$_3$</u>), 1.14 (9H, s, -O*t*-<u>Bu</u>).

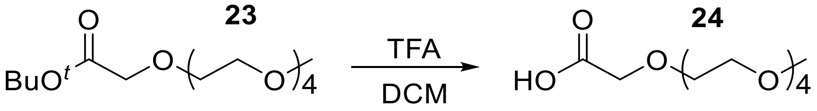

TFA (5 mL) was added to a solution of **23** (1.3 g, 4.0 mmol) in DCM (20 mL) and the mixture stirred for 5 h. The mixture was then reduced in volume to ~5 mL under vacuum and diluted with DCM (50 mL). The organics were extracted with H_2O (50 mL) and the aqueous phase concentrated under vacuum to afford the product **24** as a colourless oil (0.6 g, 2.3 mmol, 56%). Data were consistent with those previously reported.[25]

^{1}H NMR (400 MHz, $CDCl_3$) δ = 3.99 (2H, s, -C<u>H$_2$</u>COOH), 3.64 (14H, app s, PEG), 3.52 (2H, app s, PEG), 3.34 (3H, s, -OC<u>H$_3$</u>).

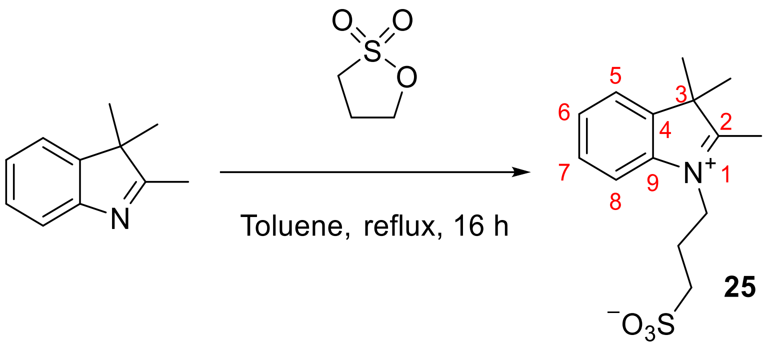

A mixture of 2,3,3-trimethylindolenine (2.00 mL, 12.7 mmol) and 1,3-propanesultone (1.55 g, 12.7 mmol) in toluene (50 mL) was refluxed for 20 h, during which time a dark red precipitate formed. After cooling to r.t., the reaction mixture was concentrated under reduced pressure. The residue was redissolved in dichloromethane (5 mL) and the solution added dropwise to diethyl ether (200 mL). The resultant precipitate was collected by filtration, washed with diethyl ether (50 mL), and dried in air to yield a red oil (3.10 g, 11.0 mmol, 87%). Data were consistent with those previously reported.[2]

^{1}H NMR (400 MHz, MeOD) δ 8.01-7.93 (m, 1H, H5), 7.78-7.70 (m, 1H, H7), 7.68-7.59 (m, 2H, H6, H8), 4.78-4.67 (m, 2H, PhCH_2), 3.03-2.93 (m, 2H, C$H_2SO_3^-$), 2.43-2.26 (m, 2H, C$H_2CH_2SO_3^-$), 1.58 (s, 6H, 2 × CH_3).

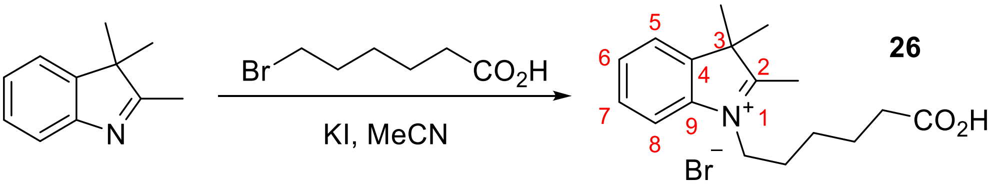

A mixture of 2,3,3-trimethylindolenine (2.00 mL, 12.7 mmol), 6-bromohexanoic acid (3.16 g, 16.2 mmol), and potassium iodide (2.69 g, 16.2 mmol) in acetonitrile (20 mL) was refluxed for 20 h. After cooling to r.t., the reaction mixture was added dropwise to diethyl ether (200 mL). The resultant precipitate was collected by filtration, washed with diethyl ether (50 mL), and dried in air to yield a red solid (4.38 g, 11.0 mmol, 87%). Data were consistent with those previously reported.[3]

^{1}H NMR (400 MHz, DMSO-d_6) δ 7.91-7.97 (1H, m, H5), 7.76-7.784 (1H, m, H7), 7.53-7.63(2H, m, H6, H8), 4.41 (2H, t, J = 7.7 Hz, -CH_2N-), 2.18 (2H, t, J = 7.2 Hz, -CH_2CO_2H), 1.80 (2H, tt, J_1 = J_2 = 7.7 Hz, -CH_2CH_2N-), 1.39-1.55 (10H, -CH_2C$H_2CH_2CO_2H$, 2 × CH_3).

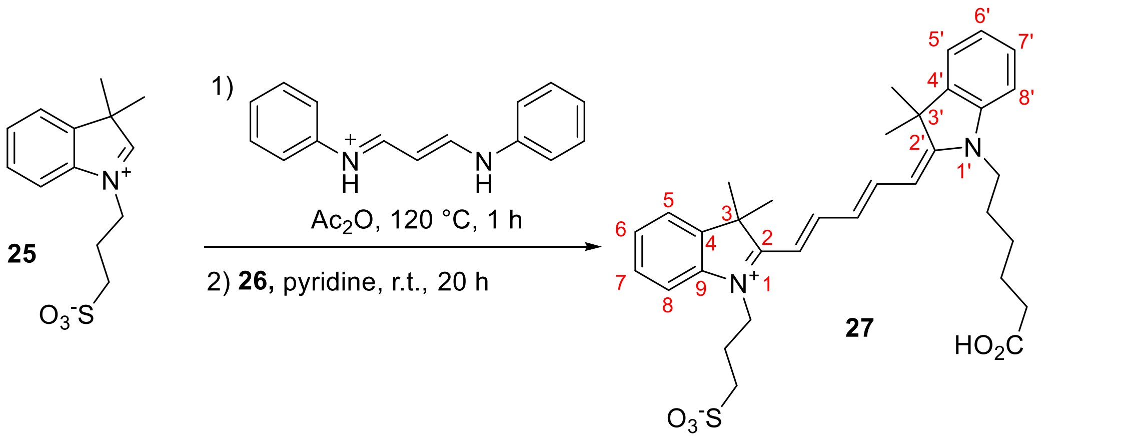

A mixture of **25** (0.5 g, 1.8 mmol) and malonaldehyde bis(phenylimine) monohydrochloride (0.47 g, 1.8 mmol) in acetic anhydride (4 mL) was heated to 120 °C for 1.5 h. After cooling to r.t, a solution of **26** (0.94 g, 2.3 mmol) in pyridine (4 mL) was added and stirring was continued at r.t. for a further 16 h. The reaction mixture was then added dropwise to diethyl ether (200 mL), and the resultant precipitate collected by filtration, washed with diethyl ether (30 mL), and dried under vacuum. The solid was then redissolved in methanol (10 mL) and concentrated under reduced pressure. The residue was purified via flash column chromatography eluting with 5-9% MeOH:DCM. Fractions containing the product were concentrated under reduced pressure to provide a blue solid (0.71 g, 1.22 mmol, 68%). Data were consistent with those previously reported.[3]

^{1}H NMR (400 MHz, DMSO-d_6) δ 8.31 (dd, $J_1 = J_2$ = 13.0 Hz, 2H, 2 × C<u>H</u>CHCN), 7.30-7.64 (6H, m, <u>H</u>5, <u>H</u>8**,** <u>H</u>7), 7.15-7.23 (2H, m, <u>H</u>6), 6.52 (1H, dd, $J_1 = J_2$ = 13.0 Hz, C<u>H</u>CHCHCN), 6.40 (1H, d, J = 13.0 Hz, C<u>H</u>CN), 6.27 (1H, d, J = 13.0 Hz, C<u>H</u>CN), 4.18-4.28 (2H, m, -C<u>H</u>$_2$N), 4.05 (2H, t, J = 7.2 Hz, -C<u>H</u>$_2$N), 2.51-2.58 (2H, m, C<u>H</u>$_2$SO$_3^-$), 2.16 (2H, t, J = 7.2 Hz, -C<u>H</u>$_2$CO$_2$H), 1.89-2.02 (4H, m, -C<u>H</u>$_2$-), 1.63 (s, 12H, CyC<u>H</u>$_3$), 1.45-1.54 (2H, m, -C<u>H</u>$_2$-), 1.26-1.35 (2H, m, -C<u>H</u>$_2$-).

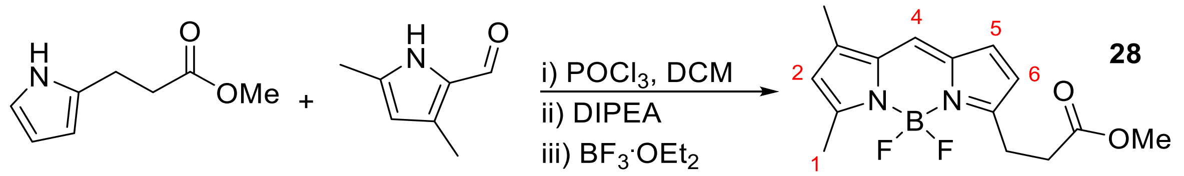

3,5-dimethylpyrrole-2-carboxaldehyde (138 mg, 1.1 mmol) was added portionwise to a solution of methyl 3-(1H-pyrrol-2-yl)propanoate (155 mg, 1.0 mmol) in DCM (10 mL) at 0 °C. After 10 min, $POCl_3$ (0.15 mL, 1.6 mmol) was added dropwise and then the reaction mixture was allowed to warm to rt and stirred for 2.5 h. The reaction mixture was then cooled to 0 °C and *N*,*N*-diisopropylethylamine (0.8 mL, 4.5 mmol) was added dropwise. After stirring at 0 °C for 30 min, $BF_3{\cdot}OEt_2$ (0.5 mL, 4.0 mmol) was added, and the reaction was allowed to warm to rt and then stirred for 17 h. The mixture was then diluted with DCM (10 mL) and brine (10 mL), filtered through a bed of Celite and the organic phase washed with brine (3 *x* 20 mL), dried over anhydrous $MgSO_4$, filtered and concentrated under vacuum. The residue was purified by flash chromatography eluting with DCM. Pure fractions were concentrated under reduced pressure to afford the product **28** as a green-red solid (150 mg, 0.49 mmol, 49%). Data were consistent with those previously reported.[20]

^{1}H NMR (500 MHz, $CDCl_3$): δ = 7.08 (1H, s, 1H, H4), 6.89 (1H, d, *J* = 4.1 Hz, H5), 6.27 (1H, d, *J* = 4.1 Hz, H6), 6.11 (1H, s, H2), 3.69 (3H, s, -OMe), 3.29 (2H, t, *J* = 7.6 Hz, -CH_2Ar), 2.77 (2H, t, *J* = 7.6 Hz, -C$H_2$$CO_2$Me), 2.56 (3H, s, H1), 2.25 (3H, s, H3).

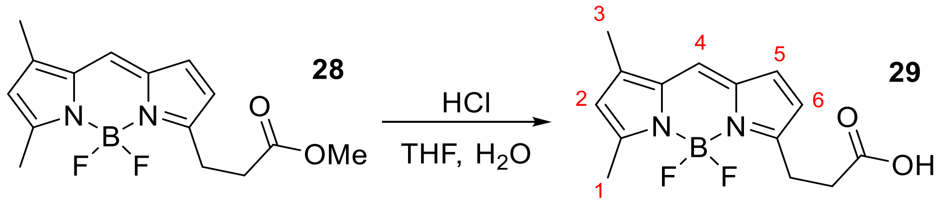

Conc. HCl (1 mL) was added to a solution of methyl ester **28** (27 mg, 88 μmol) in THF (3 mL) and water (2 mL) at 0 °C. The mixture was stirred at rt for 5 days, and then diluted with water (20 mL). The aqueous was extracted with DCM (3 × 10 mL) and the combined organics dried over $MgSO_4$, filtered, and concentrated under vacuum. The residue was purified by flash column chromatography, eluting with 1% AcOH:DCM. Pure fractions were concentrated under vacuum to afford **29** as a red crystalline solid (15 mg, 51 μmol, 58%). Data were consistent with those previously reported.[20]

^{1}H NMR (400 MHz, $CDCl_3$): δ = 7.09 (1H, s, H4), 6.87 (1H, d, *J* = 4.1 Hz, H5), 6.27 (1H, d, *J* = 4.1 Hz, H6), 6.11 (1H, s, H2), 3.29 (2H, t, *J* = 7.6 Hz, -CH_2Ar), 2.82 (2H, t, *J* = 7.6 Hz, - C$H_2$$CO_2$H), 2.56 (3H, s, H1), 2.25 ppm (3H, s, H3).

General acid chloride synthesis procedure

Oxalyl chloride (2.5 eq.) was added dropwise to a solution of carboxylic acid (1.0 eq.) and DMF (1-3 drops) in anhydrous DCM at 0 °C. The reaction was then warmed to rt and stirred for 15 min. The solvent was then removed under vacuum and the residue triturated with toluene (2 × 10 mL). The residue was then used directly in the next step without further purification or analysis.

3. NASA synthesis

General procedure A

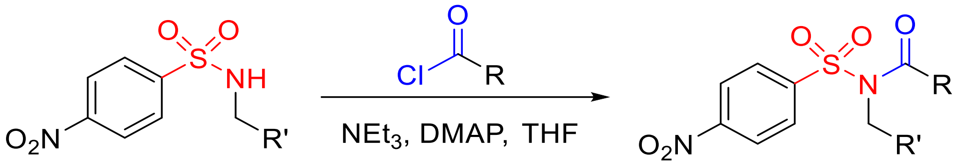

To a solution of secondary sulfonamide (1.0 eq) and acyl chloride (1.5 eq) in THF was added DMAP (10 mol%) and then triethylamine (1.5 eq) dropwise. The mixture was then stirred at rt for 17 h, then concentrated *in vacuo*. Sat. $NaHCO_3$ was added, and the mixture extracted with EtOAc (×3). The combined organics were dried over $MgSO_4$, filtered and concentrated *in vacuo*. The residue was then purified via flash column chromatography as specified below.

General procedure B

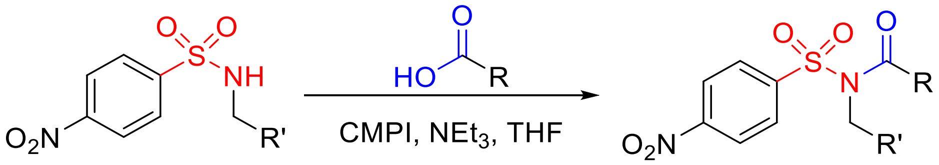

A solution of secondary sulfonamide (1.0 eq), carboxylic acid (1.5 eq) and 2-chloro-1-methylpyridinium iodide (CMPI) (1.5 eq) in THF was cooled to 0 ˚C under argon, and triethylamine (3.0 eq) was added dropwise over ~5 min. The mixture was then warmed to rt and stirred for 17 h. The mixture was then purified via aqueous work-up and subsequent flash column chromatography as specified below.

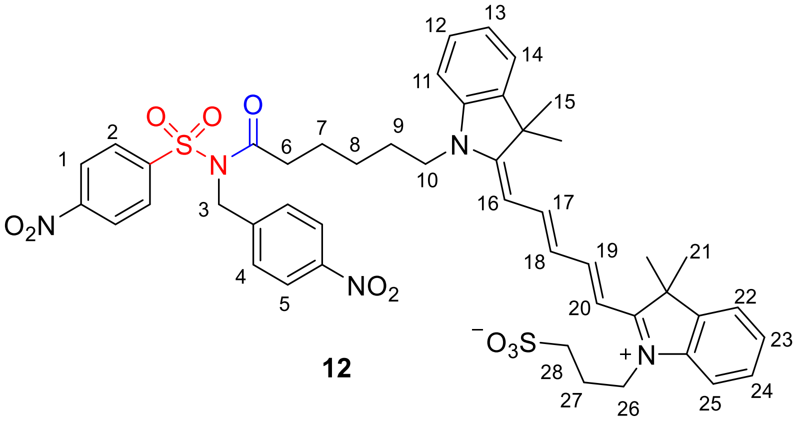

12

Produced using general procedure A on a 0.57 mmol scale. The reaction mixture concentrated *in vacuo*. Column chromatography was performed with 1-10% MeOH:DCM. **12** was obtained as a blue solid (110 mg, 0.12 mmol, 21%).

^{1}H NMR (400 MHz, $CDCl_3$) δ = (400 MHz, DMSO-d_6) δ = 8.41-8.45 (2H, m, H2), 8.22-8.37 (6H, m, H1, H5, H17, & H19), 7.19-7.63 (10H, m, H4, H11-H14, & H22-H25), 6.44-6.52 (2H, m, H16/20 & H18), 6.21 (1H, d, *J* = 13.7 Hz, H16/20), 5.31 (2H, s, H3), 4.29 (2H, t, *J* = 7.6 Hz, H10/26), 4.01 (2H, t, *J* = 6.8 Hz, H10/26), 2.58 (2H, t, *J* = 6.7 Hz, H28), 1.97-2.04 (2H, m, H6), 1.67 (6H, s, H15/21), 1.63 (6H, s, H15/21), 1.55 (2H, t, *J* = 7.5 Hz, H7/8/9), 1.46 (2H, t, *J* = 7.3 Hz, H7/8/9), 1.23-1.28 (2H, m, H7/8/9); **^{13}C NMR** (101 MHz, DMSO-D_6) δ = 173.1 (C39), 172.3 (C41), 162.7 (C40), 154.3 (C38), 153.9 (C35), 150.5 (C19), 146.9 (C29), 144.9 (C37), 144.0 (C34), 142.1 (C30), 142.0 (C36), 141.1 (C33), 141.1 (C17), 129.7 (C18), 128.5 (C24), 128.3 (C4), 127.5 (C12), 125.6 (C2), 124.8 (C23), 124.6 (C1), 124.5 (C22), 123.9 (C5), 122.4 (C14), 122.4 (C13), 111.2 (C25), 110.9 (C20), 103.6 (C11), 102.9 (C16), 49.3 (C28), 49.0 (C26), 48.8 (C3), 47.8 (C32), 43.0 (C31), 42.7 (C10), 34.7 (C6), 27.1 (C15), 27.1 (C21), 26.5 (C9), 25.1 (C8), 23.4 (C7), 23.2 (C27); **HRMS** (ESI$^+$) *m/z*: calculated for $C_{47}H_{51}N_5NaO_{10}S_2$ $[M+Na]^+$ = 932.2975; obs. = 932.3040; **IR** (film, v_{max}/cm^{-1}): 1705 (C=O), 1479 (N-O), 1374 (S=O), 1333 (S=O); **Mp**: 180 – 186 °C.

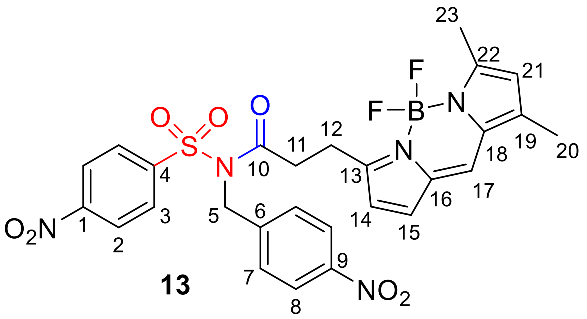

13

Produced using general procedure A on a 0.17 mmol scale. The reaction mixture was diluted with DCM (10 mL) and washed with aqueous acetic acid (3% *v/v*, 2 × 20 mL), H_2O (20 mL), sat. $NaHCO_3$ (10 mL) and brine (10 mL). Column chromatography

was performed with 20-50% EtOAc:Petrol. **13** was obtained as a red solid (62 mg, 0.11 mmol, 63%).

^{1}H NMR (400 MHz, $CDCl_3$) δ = 8.23 (2H, d, *J* = 9.0 Hz, H2), 8.17 (2H, d, *J* = 8.8 Hz, H8), 7.97 (2H, d, *J* = 9.0 Hz, H3), 7.51 (2H, d, *J* = 8.8 Hz, H7), 7.02 (1H, s, H17), 6.74 (1H, d, *J* = 4.0 Hz, H15), 6.14 (1H, s, H21), 6.05 (1H, d, *J* = 4.0 Hz, H14), 5.18 (2H, s, H5), 3.14 (2H, t, *J* = 6.9 Hz, 2H, H11), 2.99 (2H, t, *J* = 6.9 Hz, H12), 2.49 (3H, s, H23), 2.27 (3H, s, H20); **^{13}C NMR** (101 MHz, $CDCl_3$) δ = 172.1 (C10), 161.5 (C22), 155.0 (C13), 150.5 (C4), 147.6 (C6), 145.2 (C18), 144.6 (C1), 143.4 (C9), 135.5 (C20), 133.1 (C16), 129.3 (C3), 128.5 (C7), 127.4 (C15), 124.4 (C2), 124.1 (C8), 123.8 (C17), 121.1 (C21), 117.6 (C14), 49.4 (C5), 36.4 (C11), 23.7 (C12), 15.0 (C23), 11.4 (C19); **HRMS** (ESI$^+$) *m/z*: calculated for $C_{27}H_{24}BF_2N_5NaO_7S$ $[M+Na]^+$ = 634.1350; obs. = 634.1368.

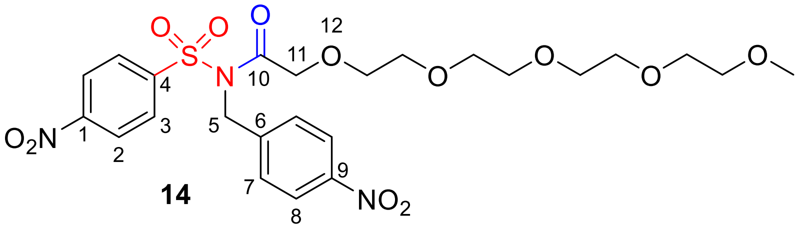

14

Produced using general procedure A on a 0.45 mmol scale. The reaction mixture was diluted with DCM (10 mL) and washed with aqueous acetic acid (3% *v/v*, 2 × 20 mL), H_2O (20 mL), sat. $NaHCO_3$ (10 mL) and brine (10 mL). Column chromatography was performed with 25-100% EtOAc:Petrol. **14** was obtained as a light yellow oil (95 mg, 0.18 mmol, 40%).

^{1}H NMR (400 MHz, $CDCl_3$) δ = 8.38 (2H, d, *J* = 8.9 Hz, H2), 8.20 (2H, d, *J* = 8.7 Hz, H8), 8.10 (2H, d, *J* = 8.9 Hz, H3), 7.55 (2H, d, *J* = 8.7 Hz, H8), 5.12 (2H, s, H5), 4.42 (2H, s, 2H, H11), 3.63-3.55 (14H, m, PEG-CH$_2$), 3.52-3.49 (2H, m, PEG-CH$_2$), 3.33 (3H, s, -CH$_3$). **^{13}C NMR** (101 MHz, $CDCl_3$) δ = 170.0 (C10), 150.9 (C1), 147.7 (C6), 144.1 (C4), 143.0 (C9), 129.5 (C2), 128.6 (C7), 124.6 (C3), 124.1 (C8), 71.9 (PEG), 71.6 (C11), 71.1 (PEG), 70.6 (PEG), 70.6 (PEG), 70.6 (PEG), 70.6 (PEG), 70.5 (PEG), 59.1 (C5), 49.1 (-CH$_3$); **HRMS** (ESI$^+$) *m/z*: calculated for $C_{24}H_{31}NaN_3O_{12}S$ $[M+Na]^+$: 608.1521; obs. = 608.1522.

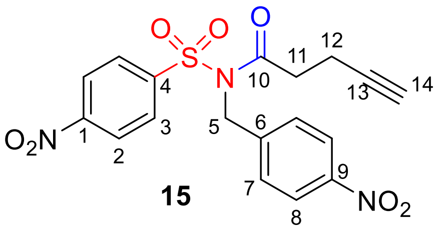

15

Produced using general procedure B on a 0.30 mmol scale. Column chromatography was performed with 10-20% EtOAc:Petrol. **15** was obtained as a white solid (50 mg, 0.12 mmol, 40%). **^{1}H NMR** (400 MHz, Acetone-d_6) δ = 8.48 (2H, d, *J* = 9.0 Hz, H2), 8.32 (2H, d, *J* = 8.9 Hz, H8), 8.27 (2H, d, *J* = 9.0 Hz, H3), 7.69 (2H, d, *J* = 8.9 Hz, H7), 5.40 (2H, s, H5), 2.95 (2H, t, *J* = 7.1 Hz, H11), 2.39 (2H, td, *J* = 7.1, 2.7 Hz, H12), 2.33 (1H, t, *J* = 2.7 Hz, H14); **^{13}C NMR** (101 MHz, Acetone-d_6) δ = 172.2 (C10), 151.9 (C1), 148.4 (C9), 145.4 (C4), 145.4 (C6), 130.7 (C2), 128.9 (C8), 125.3 (C3), 124.6 (C7), 83.1 (C13), 70.6 (C14), 50.2 (C5), 36.0 (C11), 14.3 (C12); **HRMS** (ESI$^+$) *m/z*: calculated for $C_{18}H_{15}N_3NaO_7S$ [M+Na]$^+$ = 440.0528; obs. = 440.0523; **IR** (film, ν_{max}/cm^{-1}): 3278 (C-H$_{alkyne}$), 1703 (C=O); **M.p.:** 142 – 144 °C.

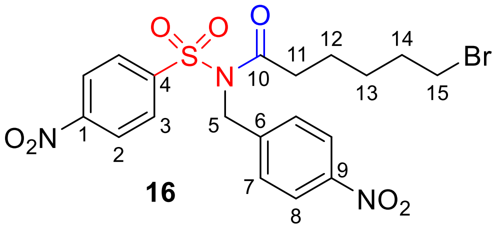

16

Produced using general procedure A on a 1.5 mmol scale. Column chromatography was performed with 10-20% EtOAc:Petrol. **16** was obtained as a white solid (740 mg, 1.44 mmol, 96%). **^{1}H NMR** (400 MHz, DMSO-d_6) δ = 8.44 (2H, d, *J* = 8.9 Hz, H2), 8.28 (2H, d, *J* = 8.8 Hz, H8), 8.26 (2H, d, *J* = 8.9 Hz, H3), 7.60 (2H, d, *J* = 8.8 Hz, H7), 5.31 (2H, s, H5), 3.41 (2H, t, *J* = 6.7 Hz, H15), 2.52 (2H, t, *J* = 7.1 Hz, H11), 1.60-1.68 (2H, m, H14), 1.37-1.44 (2H, m, H12), 1.17-1.25 (2H, m, H13); **^{13}C NMR** (101 MHz, DMSO-d_6) δ = 173.1 (C10), 150.5 (C2), 146.9 (C9), 144.9 (C4), 144.0 (C6), 129.7 (C2), 127.6 (C8), 124.6 (C3), 124.0 (C7), 49.3 (C5), 34.8 (C15), 34.7 (C7), 31.8 (C14), 26.6 (C12), 22.8 (C13); **HRMS** (ESI$^+$) *m/z*: calculated for $C_{19}H_{20}{}^{79}BrN_3NaO_7S$ [M+Na]$^+$ = 536.0103; obs. = 536.0098; **IR** (film, ν_{max}/cm^{-1}): 2943 (C-H), 1703 (C=O), 600 (C-Br); **M.p.:** 143 – 147 °C.

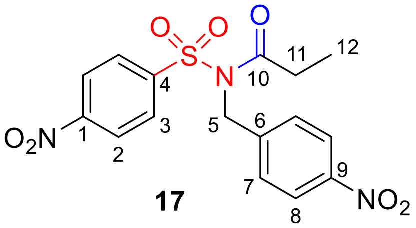

17

Produced using general procedure A on a 0.3 mmol scale. Column chromatography was performed with 10-20% EtOAc:Petrol. **17** was obtained as a yellow oil (113 mg, 0.29 mmol, 96%). **^{1}H NMR** (400 MHz, Acetone-d_6) δ = 8.47 (2H, d, *J* = 8.9 Hz, H2), 8.31 (2H,

d, J = 8.9 Hz, H8), 8.28 (2H, d, J = 8.9 Hz, H3), 7.68 (2H, d, J = 8.9 Hz, H7), 5.38 (2H, s, H5), 2.67 (2H, q, J = 7.2 Hz, H11), 0.96 (3H, t, J = 7.2 Hz, H12); **^{13}C NMR** (101 MHz, Acetone-d_6) δ = 174.5 (C10), 151.8 (C1), 148.4 (C9), 145.8 (C4), 145.7 (C6), 130.7 (C2), 128.9 (C8), 125.3 (C3), 124.7 (C7), 50.3 (C5), 29.5 (C11), 8.7 (C12); **HRMS** (ESI$^+$) *m/z*: calculated for $C_{16}H_{15}N_3NaO_7S$ $[M+Na]^+$ = 416.0528; obs. = 416.0529; **IR** (film, v_{max}/cm^{-1}): 2942 (C-H), 1703 (C=O).

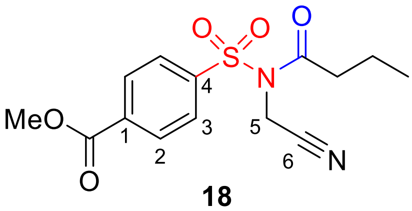

18

Produced using general procedure A on a 0.2 mmol scale. The reaction mixture was diluted with DCM (10 mL) and washed with H_2O (10 mL) and brine (10 mL). Column chromatography was performed with 20% EtOAc:Petrol. **18** was obtained as a colourless oil (40 mg, 125 µmol, 62%).

^{1}H NMR (400 MHz, $CDCl_3$) δ = 8.27 (2H, d, J = 8.7 Hz, H3), 8.06 (2H, d, J = 8.7 Hz, H2), 4.79 (2H, s, H5), 3.98 (3H, s, H12), 2.62 (2H, t, J = 7.1 Hz, H8), 1.63 – 1.58 (2H, m, H9), 0.86 (3H, t, J = 7.4 Hz, H10); **^{13}C NMR** (101 MHz, $CDCl_3$) δ 171.8 (C11), 165.1 (C7), 142.1 (C4), 135.9 (C1), 131.0 (C3), 127.9 (C2), 114.7 (C6), 53.1 (C12), 38.0 (C8), 33.2 (C5), 18.0 (C9), 13.5 (C10); **HRMS** (ESI$^+$) *m/z*: calculated for $C_{14}H_{16}N_2NaO_5S$ $[M+Na]^+$: 347.0672; obs. = 347.0672; **IR** (film, v_{max}/cm^{-1}): 1718 (C=O amide).

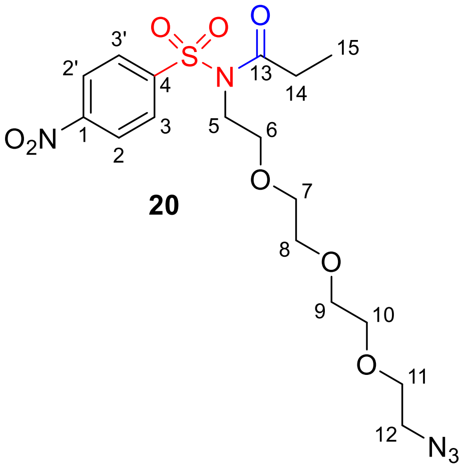

20

Produced using general procedure A on a 0.28 mmol scale. Column chromatography was performed with 10-40% EtOAc:Petrol. **20** was obtained as a brown oil (110 mg, 0.24 mmol, 86%). **^{1}H NMR** (400 MHz, $CDCl_3$) δ = 8.34 (2H, d, J = 9.0 Hz, H2), 8.19 (2H, d, J = 9.0 Hz, H3), 4.09 (2H, t, J = 5.3 Hz, H11), 3.77 (2H, t, J = 5.3 Hz, H6), 3.59-3.67 (10H, m, H7-H10, & H12), 3.33-3.39 (2H, m, H5), 2.58 (2H, q, J = 7.2 Hz, H14), 1.01 (3H, t, J = 7.2 Hz, H15); **^{13}C NMR** (101 MHz, $CDCl_3$) δ = 181.0 (C14), 156.9 (C1), 152.0 (C4), 136.2 (C2), 130.6 (C3), 84.0 (C12), 83.6 (C7), 83.3 (C11), 77.2 (C10), 76.6 (C9), 76.3 (C8), 57.2 (C13), 52.7 (C6), 35.7 (C15), 14.8 (C16); **HRMS** (ESI$^+$) *m/z*: calculated for

$C_{17}H_{25}N_5NaO_8S$ [M+Na]$^+$ = 482.1322; obs. = 482.1316; **IR** (film, ν_{max}/cm^{-1}): 2875 (C-H), 1705 (C=O).

4. References

1 T. Deng, X. Mao, Y. Li, S. Bo, Z. Yang and Z. X. Jiang, *Bioorg. Med. Chem. Lett.*, 2018, **28**, 3502–3505.

2 Z. Shi, P. Peng, D. Strohecker and Y. Liao, *J. Am. Chem. Soc.*, 2011, **133**, 14699–14703.

3 E. Herbst and D. Shabat, *Org. Biomol. Chem.*, 2016, **14**, 3715–3728.

4 L. Raynal, J. Nabarro, L. Miller, A. Dowle, S. Moul, S. Johnson, M. Fascione and C. Spicer, *ChemRxiv*, 2024, preprint, DOI: 10.26434/chemrxiv-2024-qskp0.

400 MHz

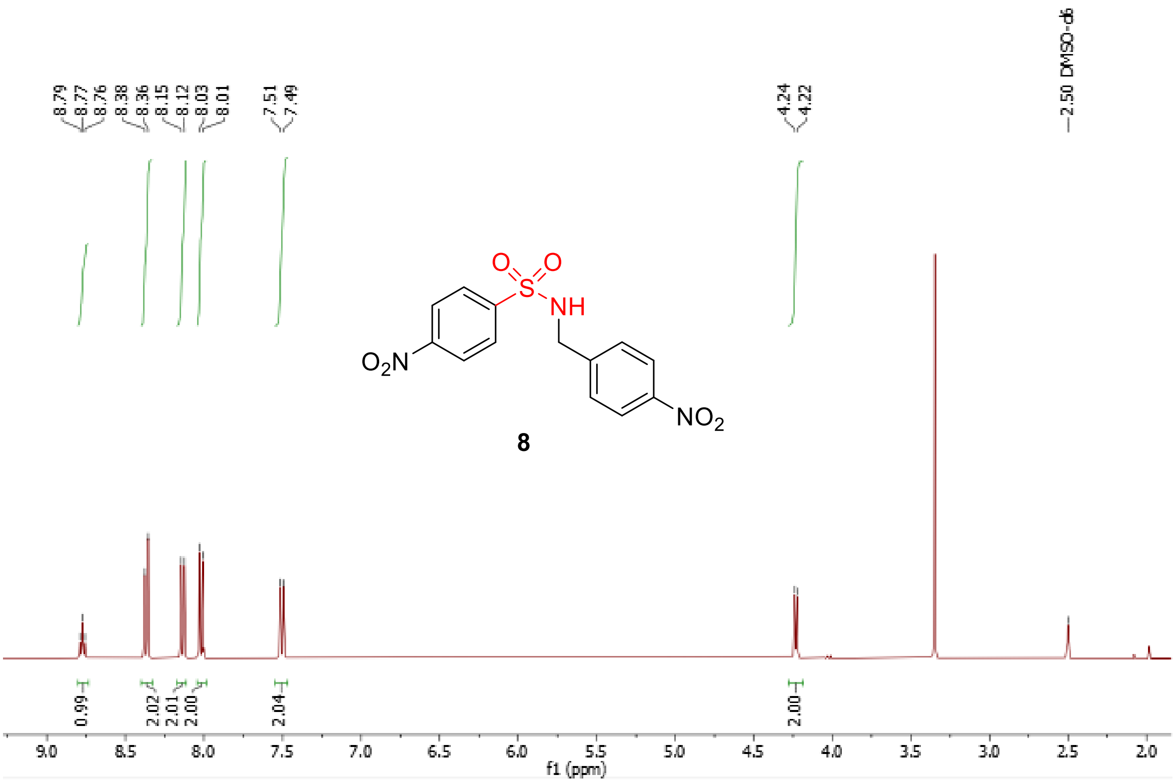

101 MHz

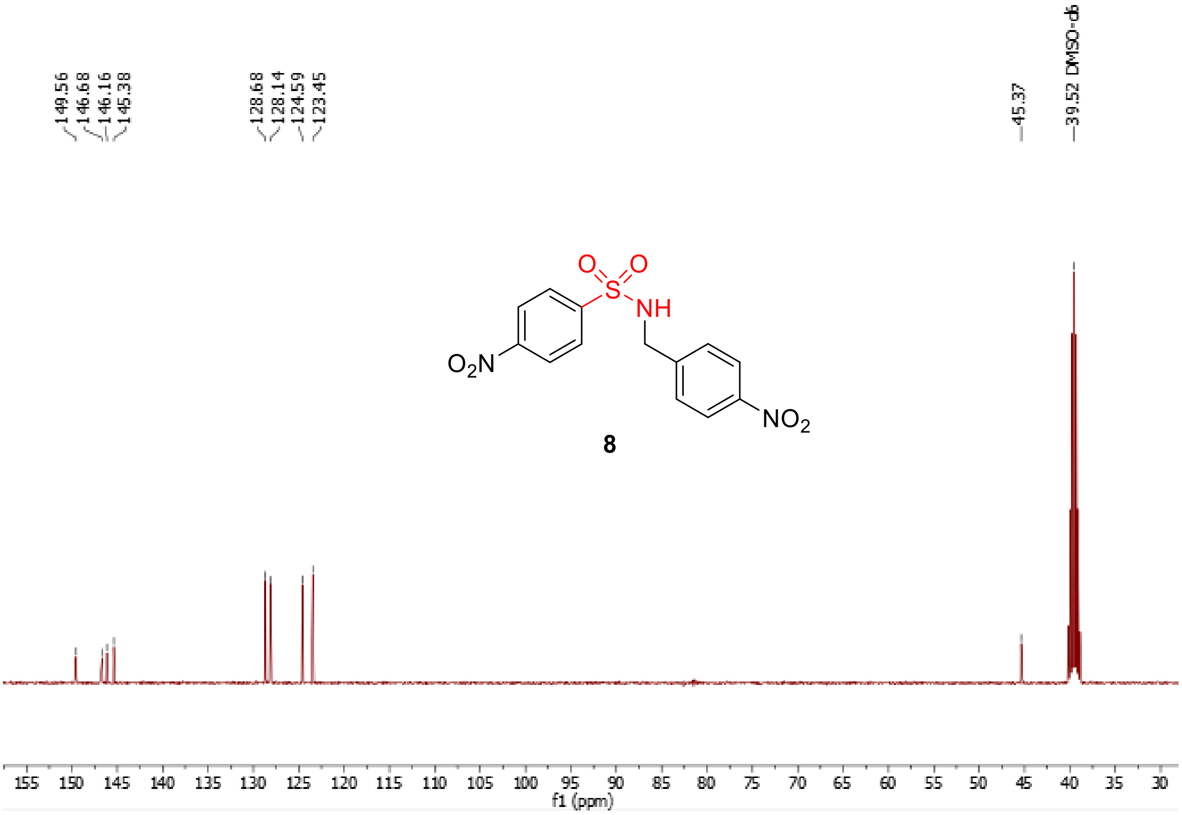

400 MHz

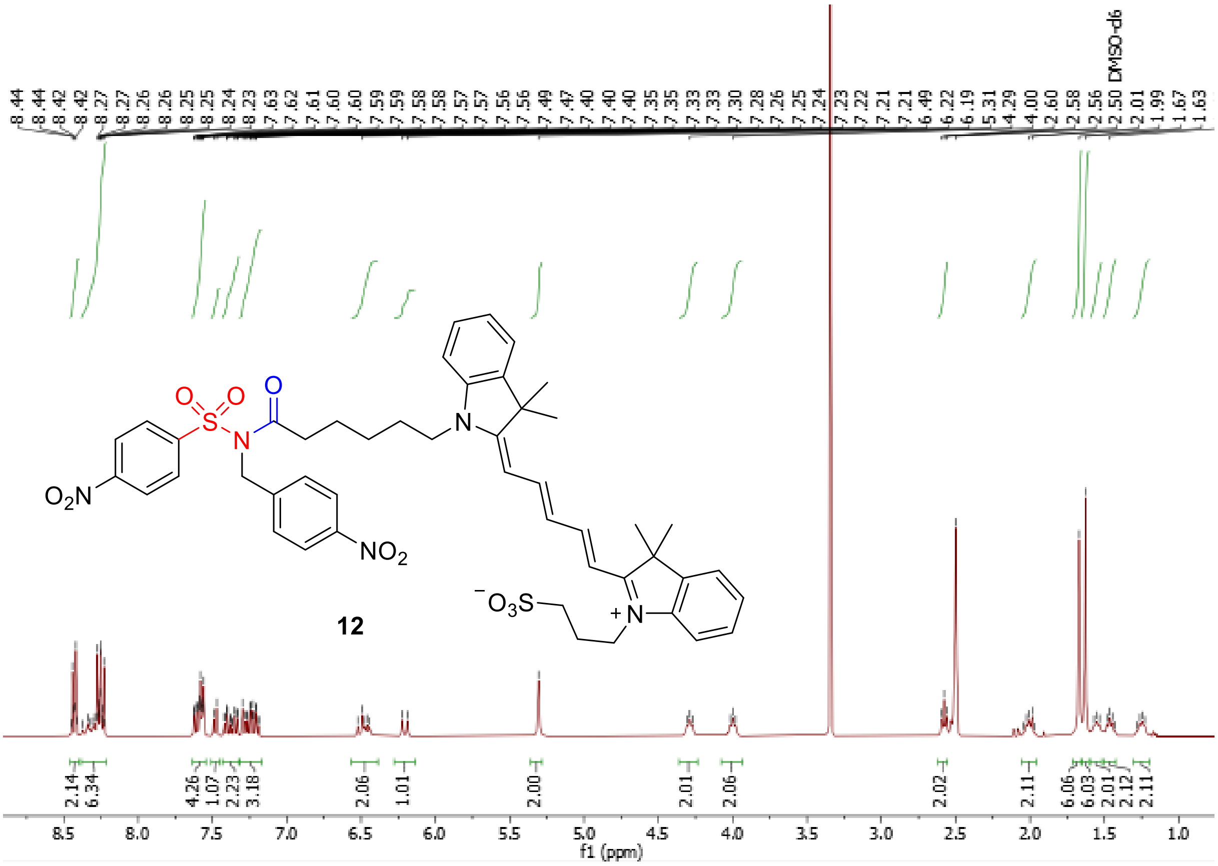

100 MHz

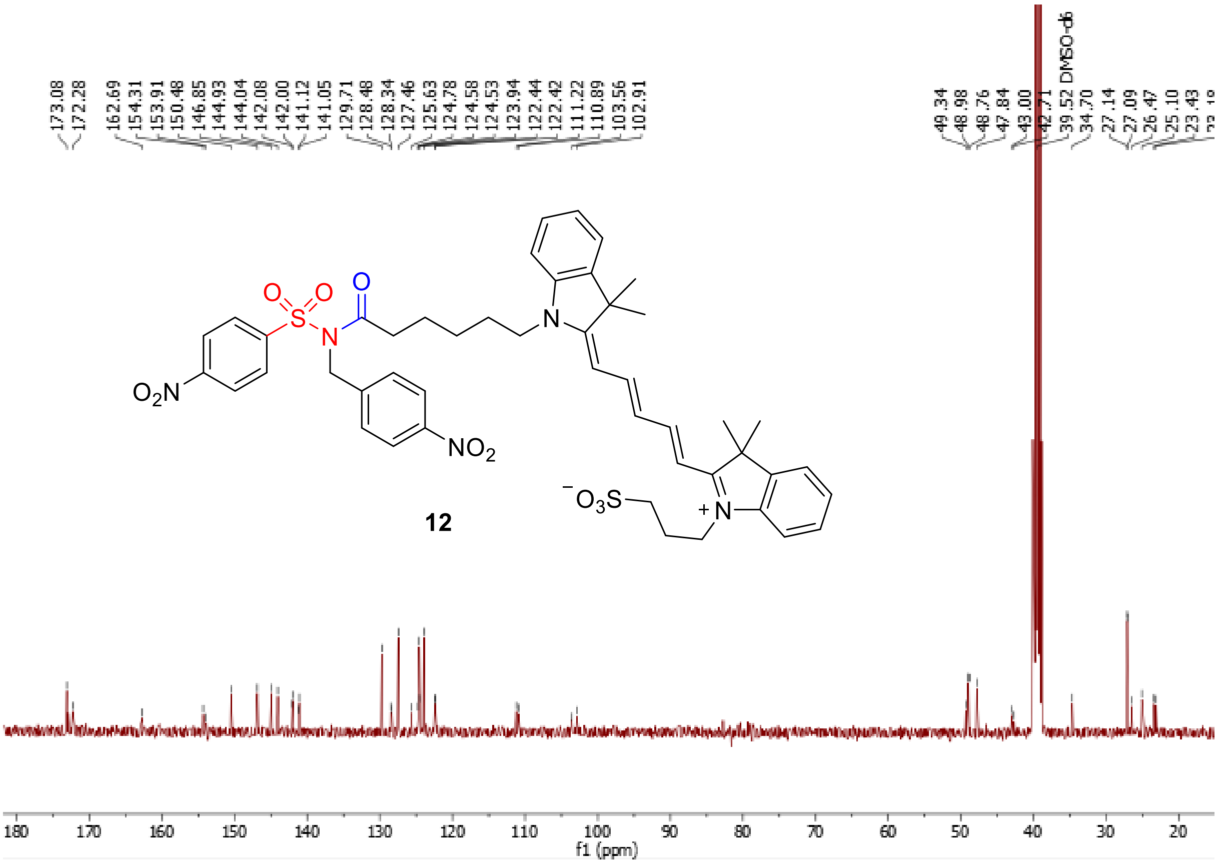

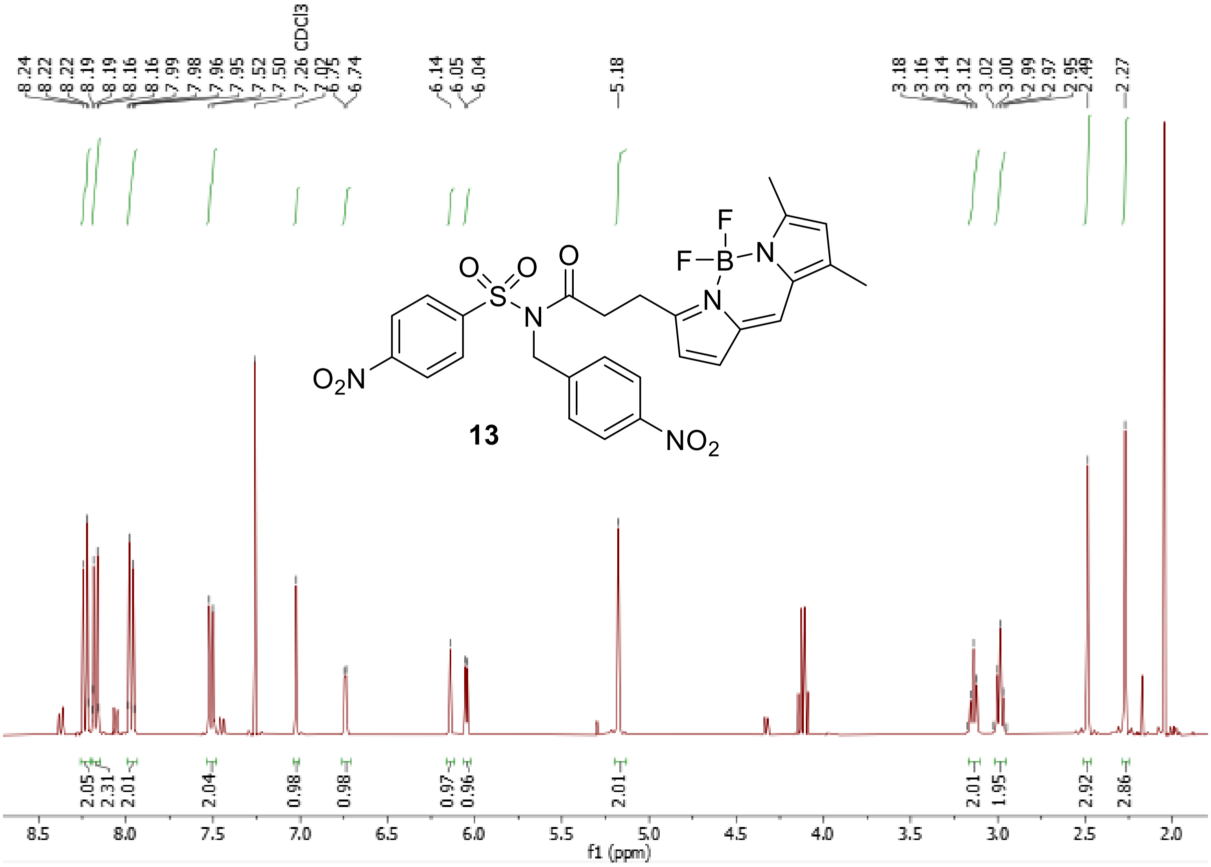
8.24 8.22 8.22 8.19 8.19 8.16 8.16 7.99 7.98 7.96 7.95 7.52 7.50 7.26 CDCl3 7.02 6.75 6.74
6.14 6.05 6.04
5.18
3.18 3.16 3.14 3.12 3.02 3.00 2.99 2.97 2.95 2.49
2.27
13
2.05 2.31 2.01 2.04 0.98 0.98 0.97 0.96 2.01 2.01 1.95 2.92 2.86
8.5 8.0 7.5 7.0 6.5 6.0 5.5 5.0 4.5 4.0 3.5 3.0 2.5 2.0
f1 (ppm)

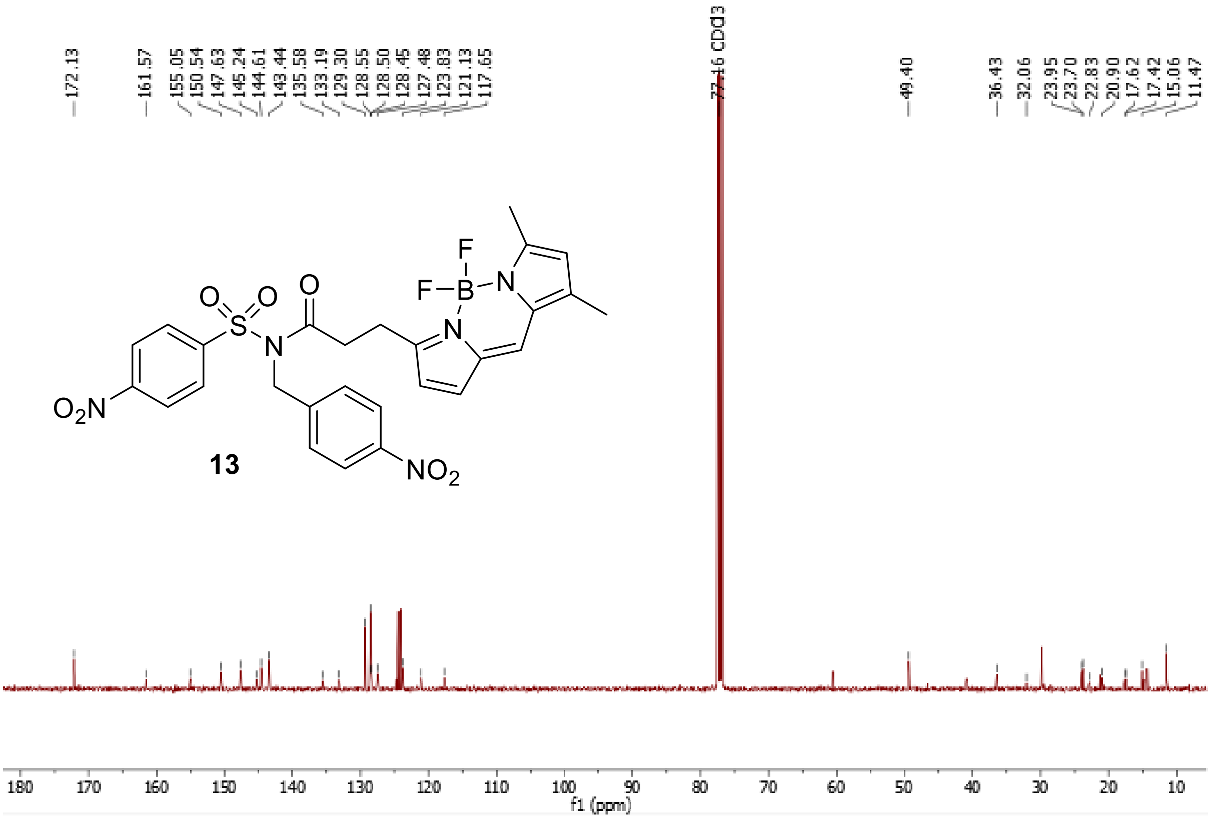
172.13
161.57 155.05 150.54 147.63 145.24 144.61 143.44 135.58 133.19 129.30 128.55 128.50 128.45 127.48 123.83 121.13 117.65
77.16 CDCl3
49.40
36.43 32.06 23.95 23.70 22.83 20.90 17.62 17.42 15.06 11.47
13
180 170 160 150 140 130 120 110 100 90 80 70 60 50 40 30 20 10
f1 (ppm)

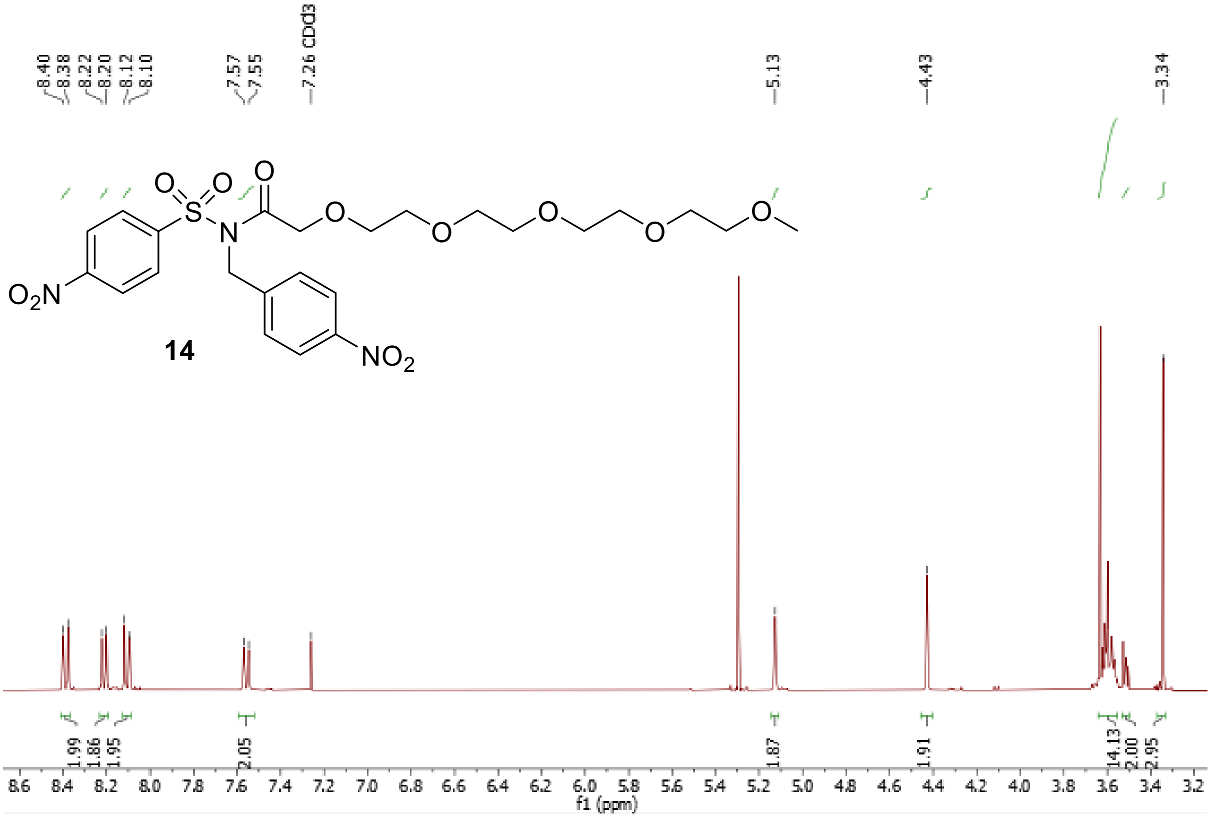

8.40
8.38
8.22
8.20
8.12
8.10
7.57
7.55
7.26 CDCl3
5.13
4.43
3.34
14
1.99
1.86
1.95
2.05
1.87
1.91
14.13
2.00
2.95
f1 (ppm)

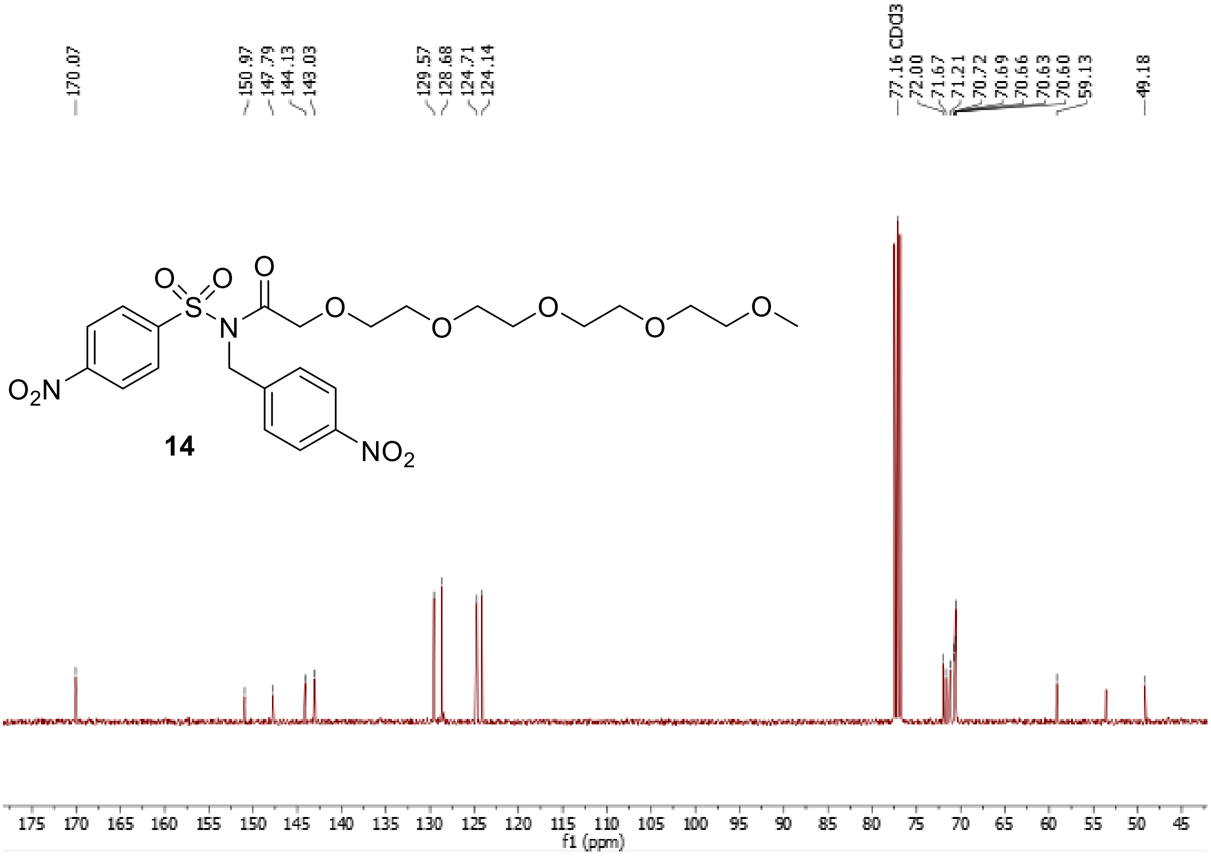

170.07
150.97
147.79
144.13
143.03
129.57
128.68
124.71
124.14
77.16 CDCl3
72.00
71.67
71.21
70.72
70.69
70.66
70.63
70.60
59.13
49.18
14
f1 (ppm)

400 MHz

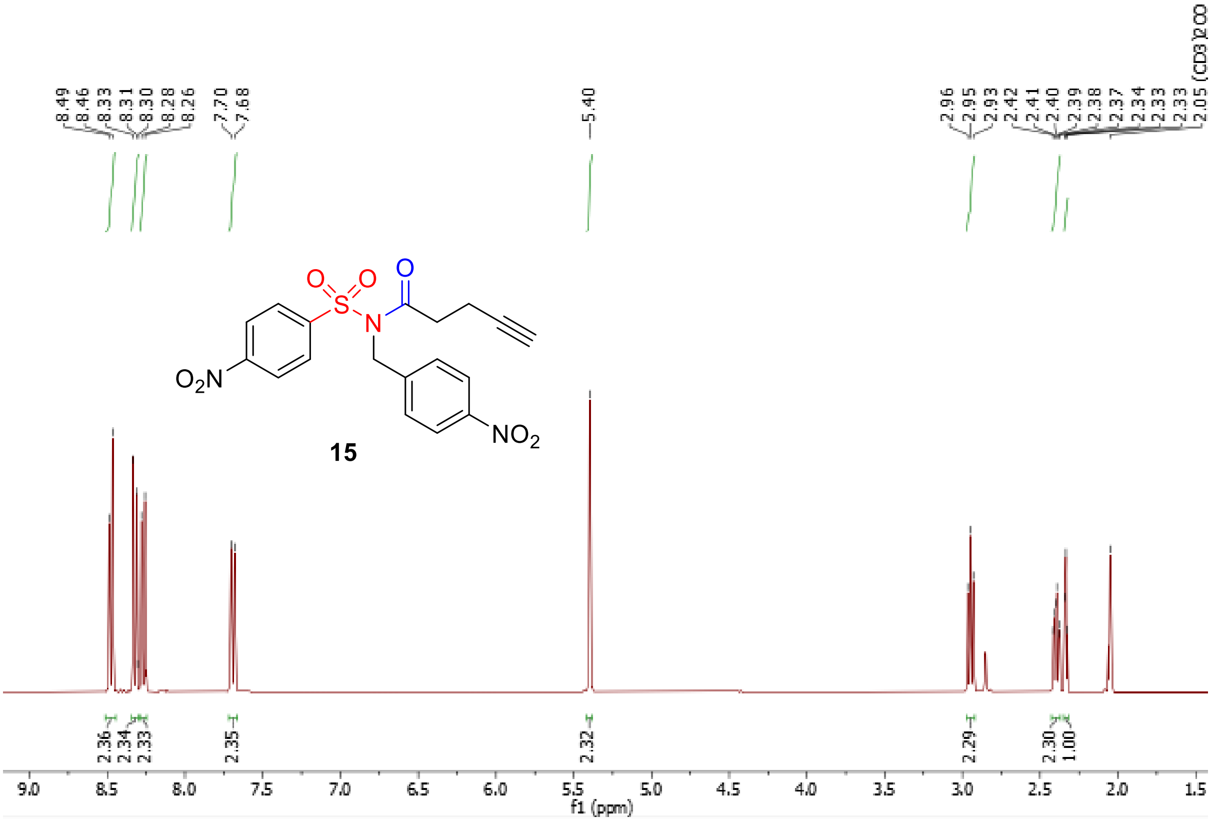

100 MHz

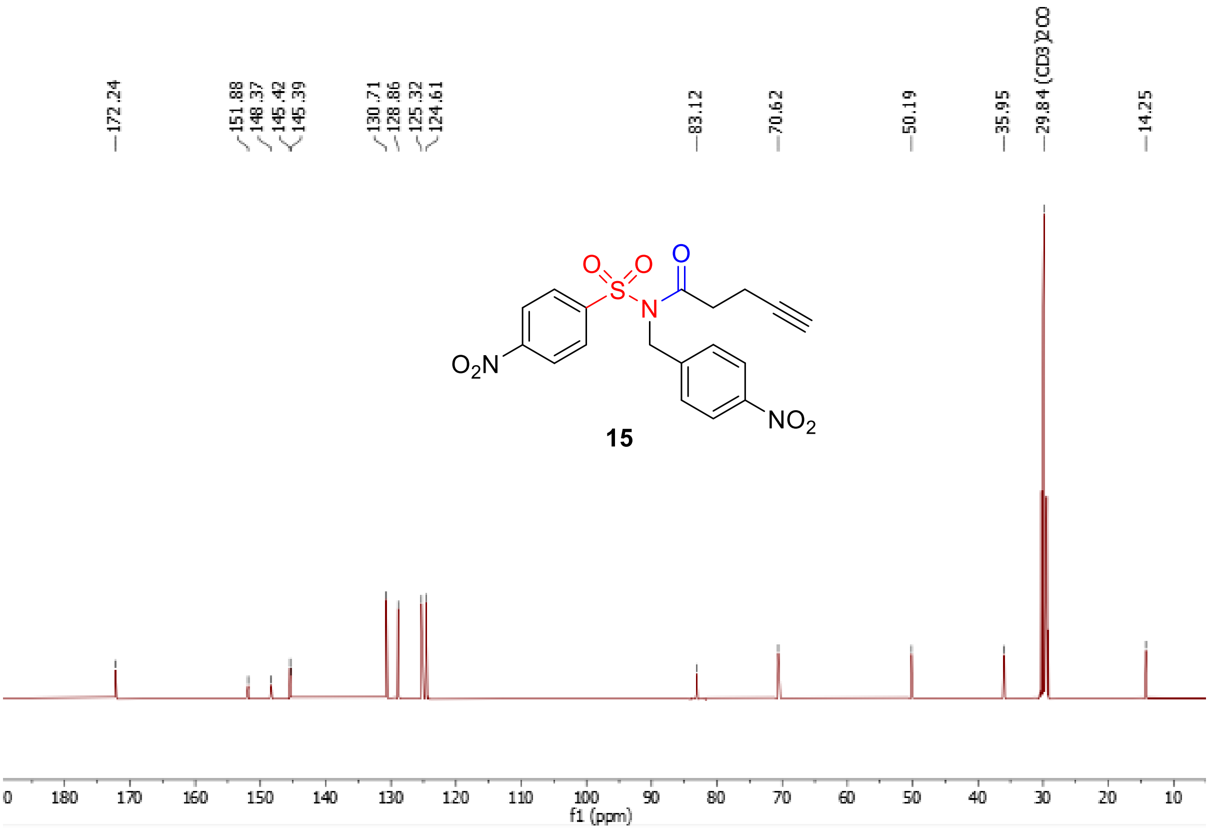

400 MHz

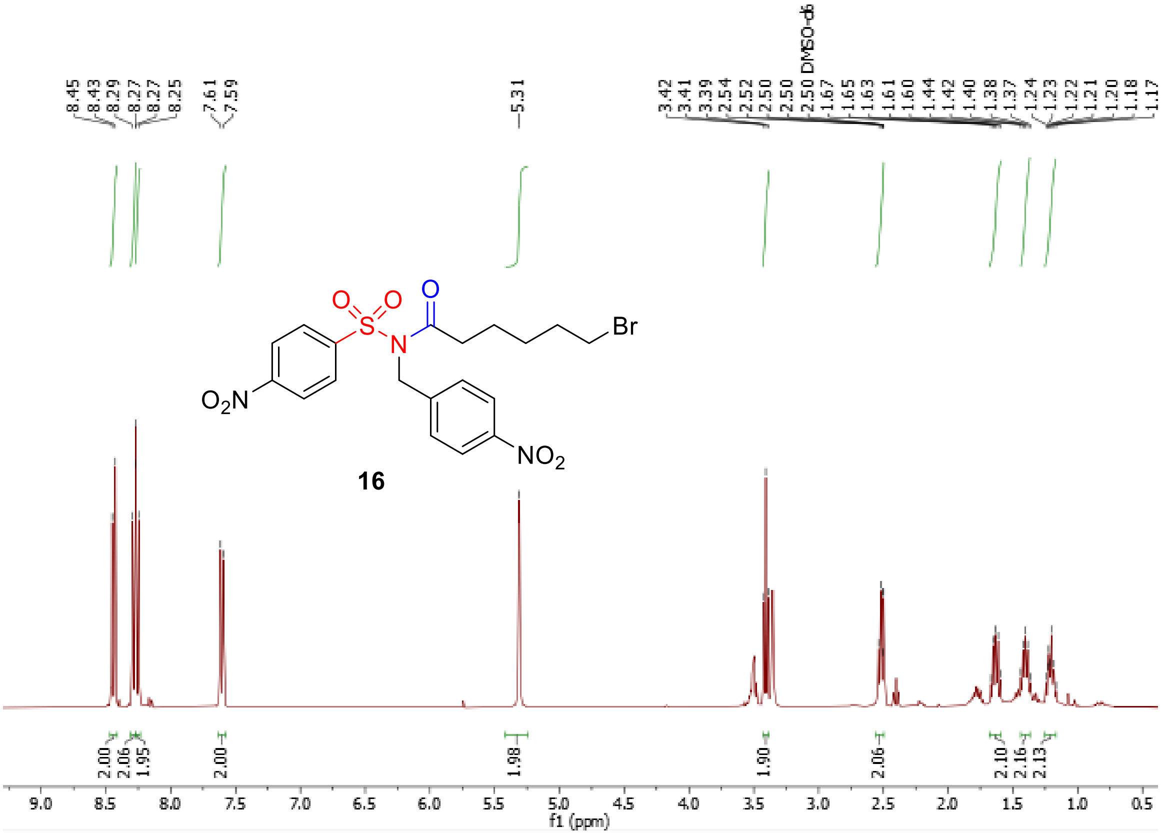
8.45
8.43
8.29
8.27
8.27
8.25
7.61
7.59
5.31
3.42
3.41
3.39
2.54
2.52
2.50
2.50
2.50 DMSO-d6
1.67
1.65
1.63
1.61
1.60
1.44
1.42
1.40
1.38
1.37
1.24
1.23
1.22
1.21
1.20
1.18
1.17
O
O
S
N
O
Br
O_2N
NO_2
16
2.00
2.06
1.95
2.00
1.98
1.90
2.06
2.10
2.16
2.13
9.0
8.5
8.0
7.5
7.0
6.5
6.0
5.5
5.0
4.5
4.0
3.5
3.0
2.5
2.0
1.5
1.0
0.5
f1 (ppm)

100 MHz

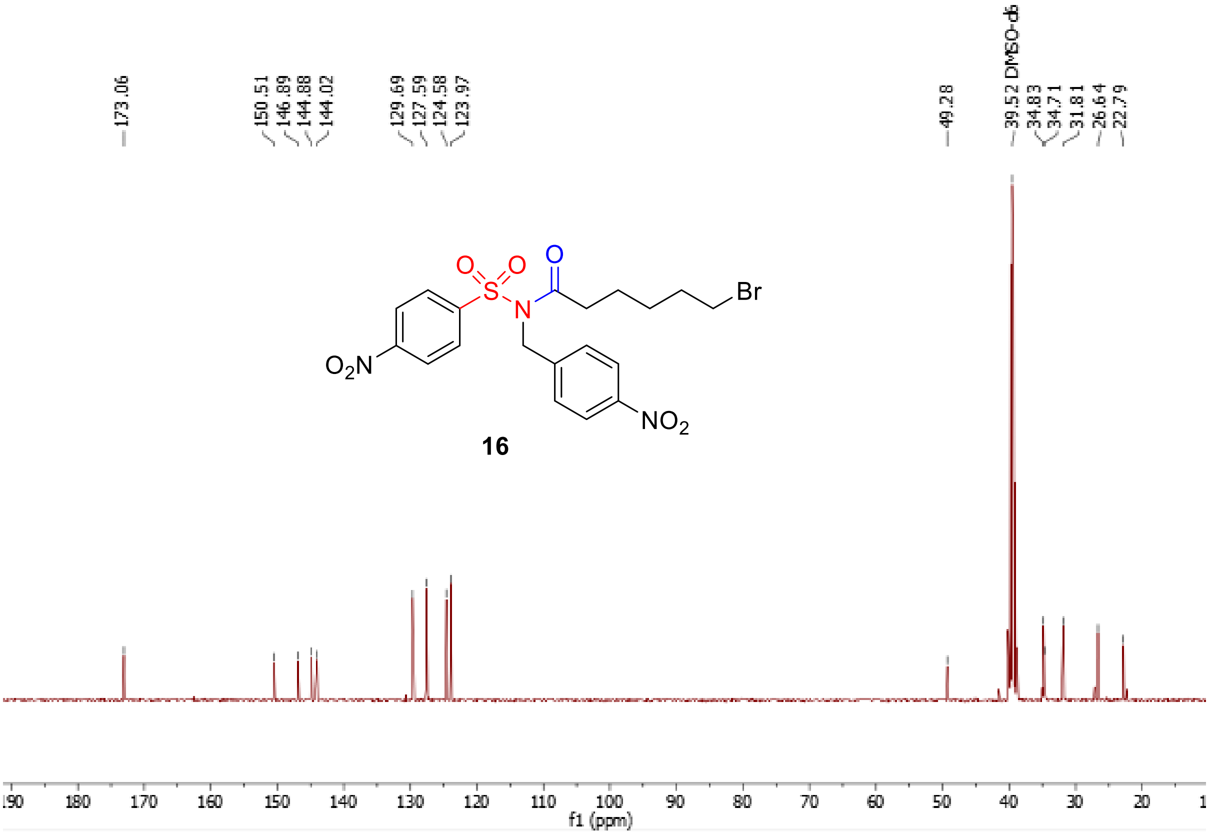
173.06
150.51
146.89
144.88
144.02
129.69
127.59
124.58
123.97
49.28
39.52 DMSO-d6
34.83
34.71
31.81
26.64
22.79
O
O
S
N
O
Br
O_2N
NO_2
16
180
170
160
150
140
130
120
110
100
90
80
70
60
50
40
30
20
f1 (ppm)

400 MHz

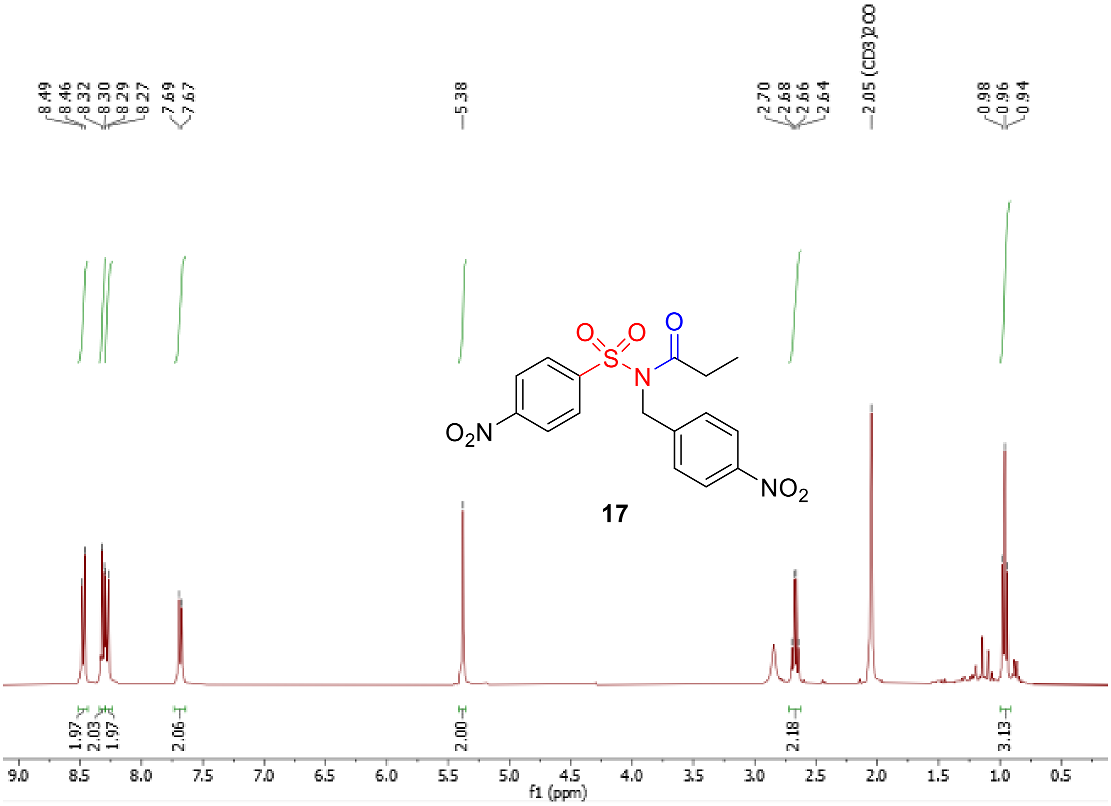

101 MHz

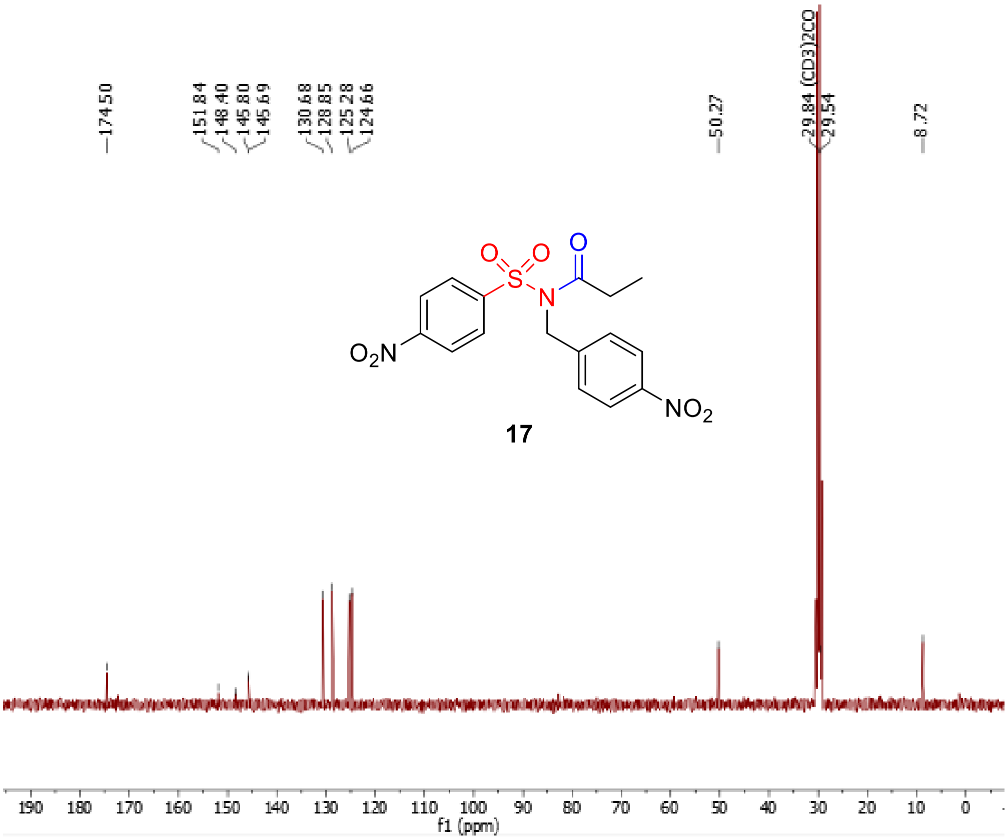

400 MHz

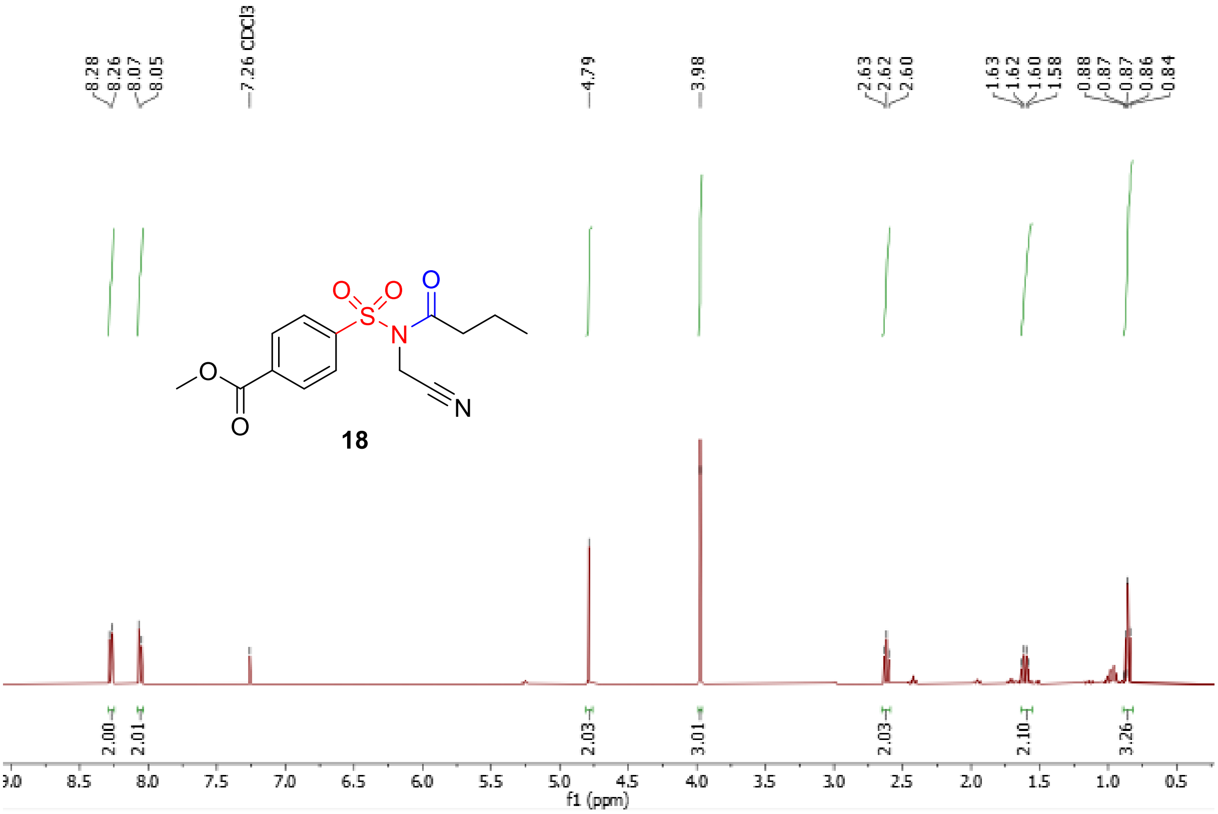

101 MHz

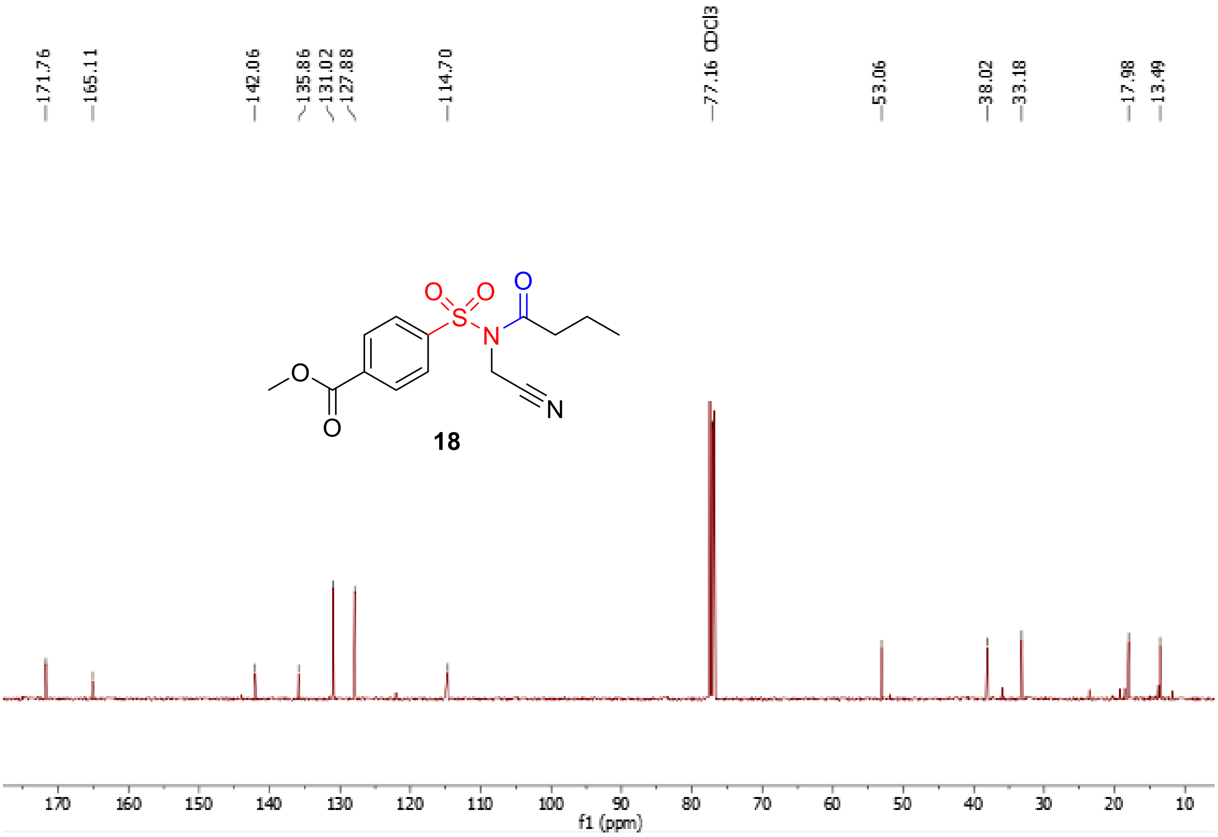

400 MHz

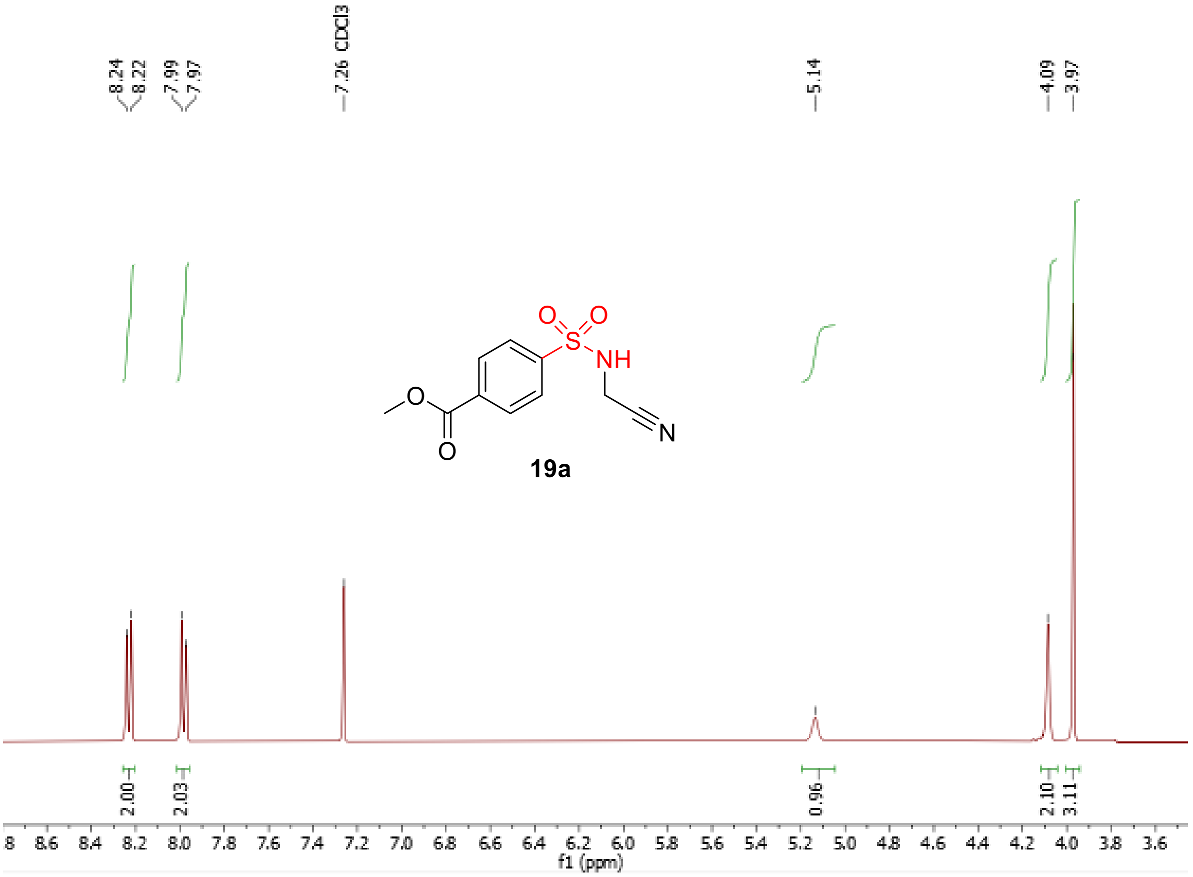

101 MHz

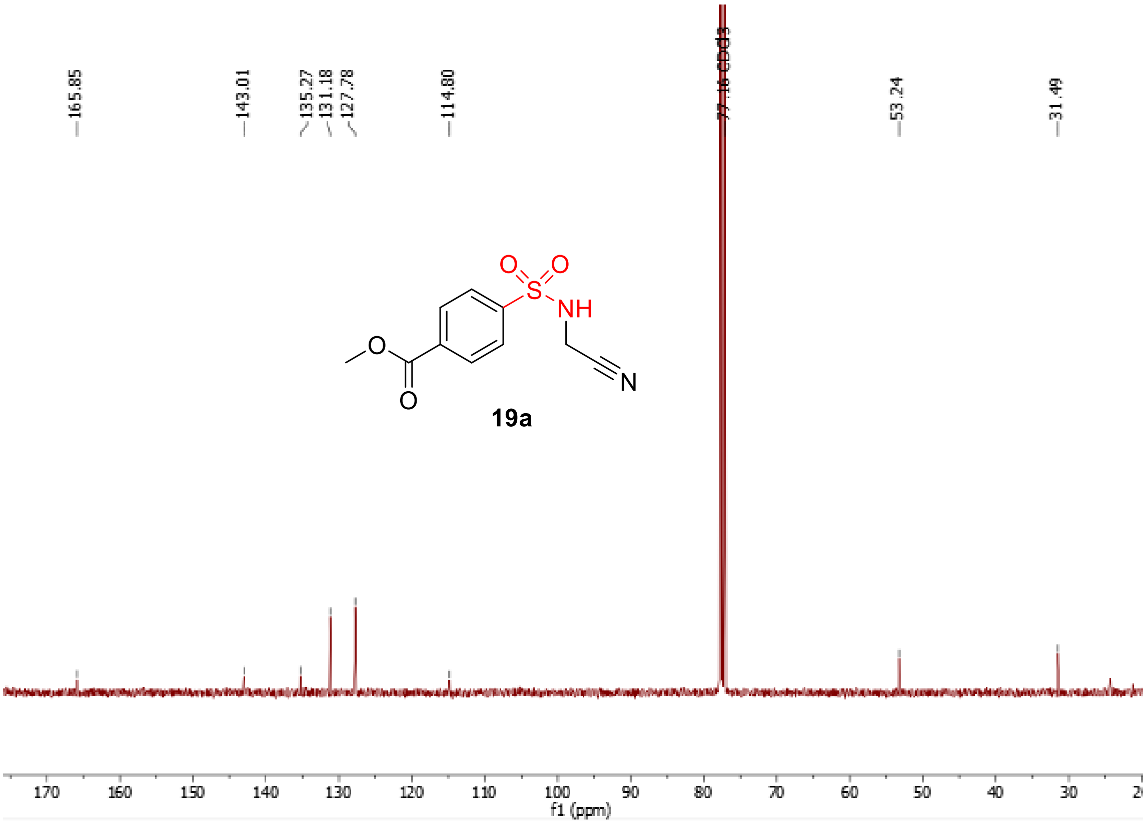

400 MHz

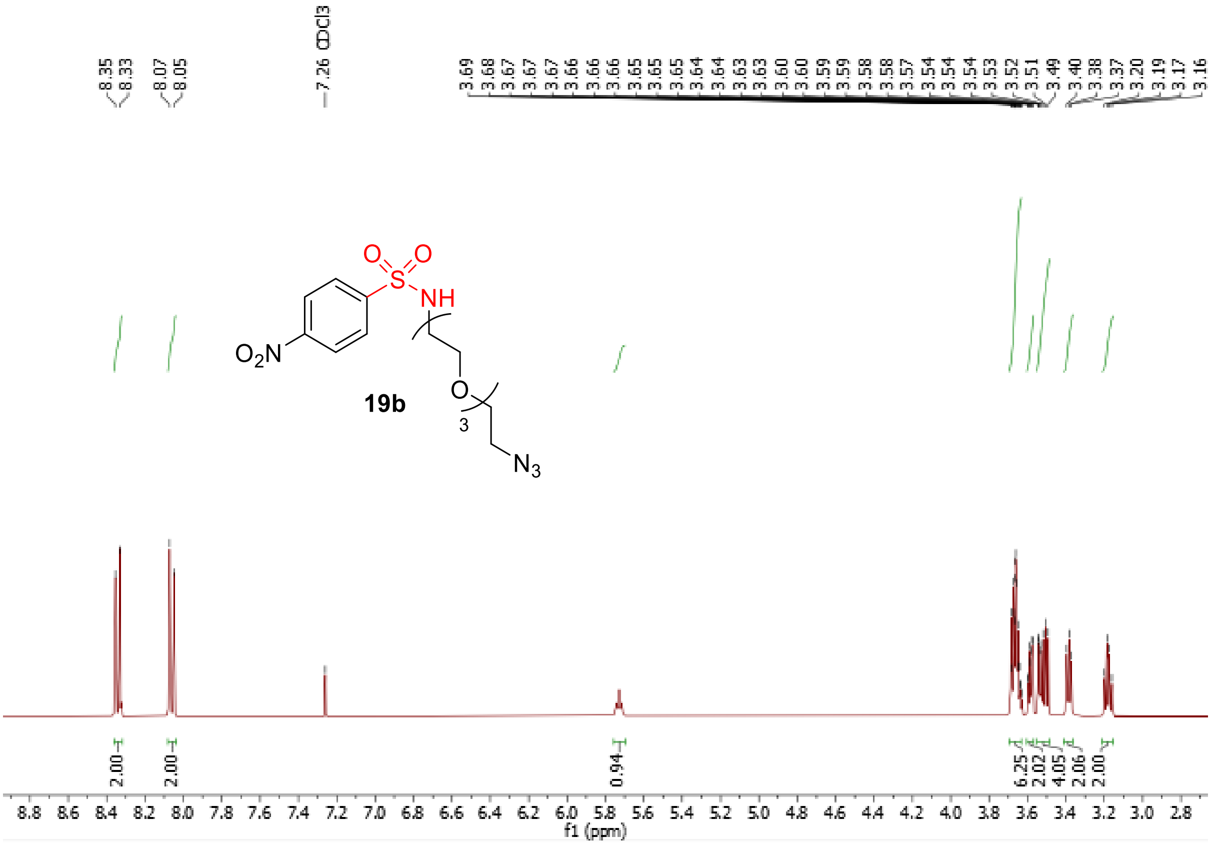

101 MHz

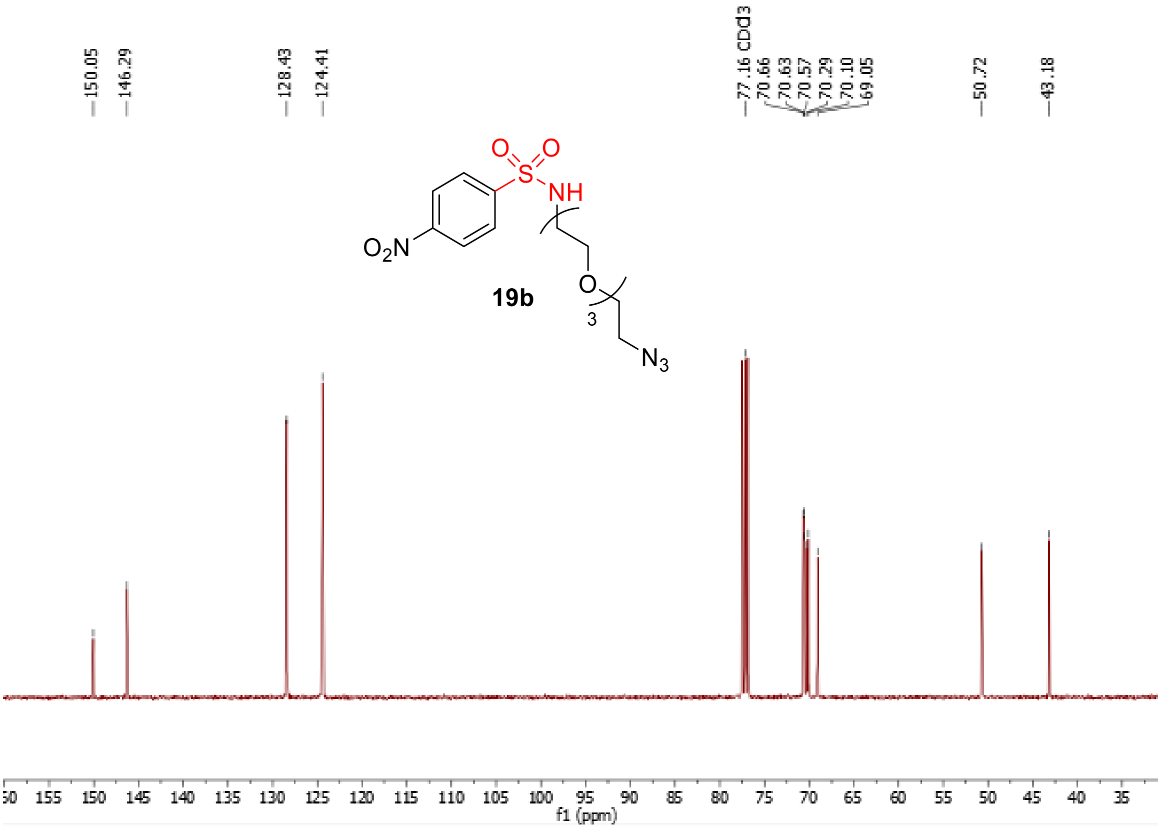

400 MHz

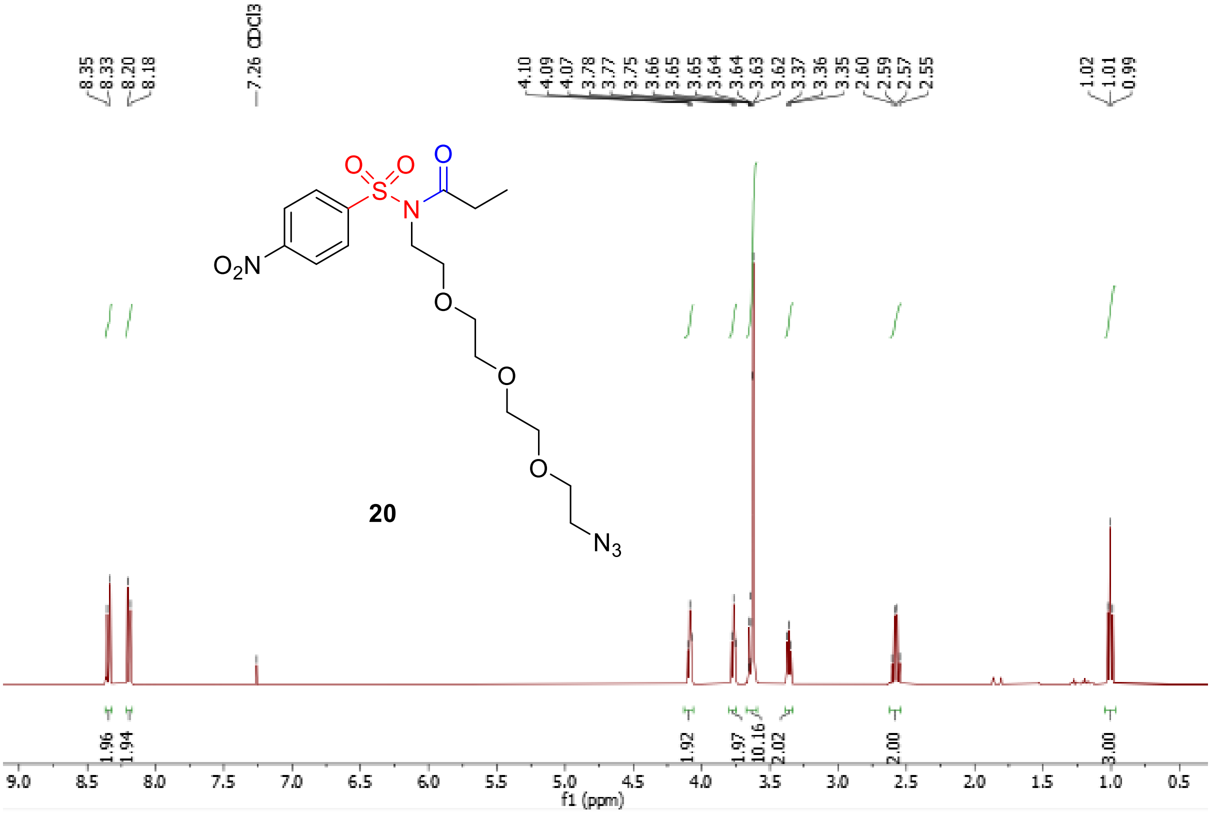

100 MHz

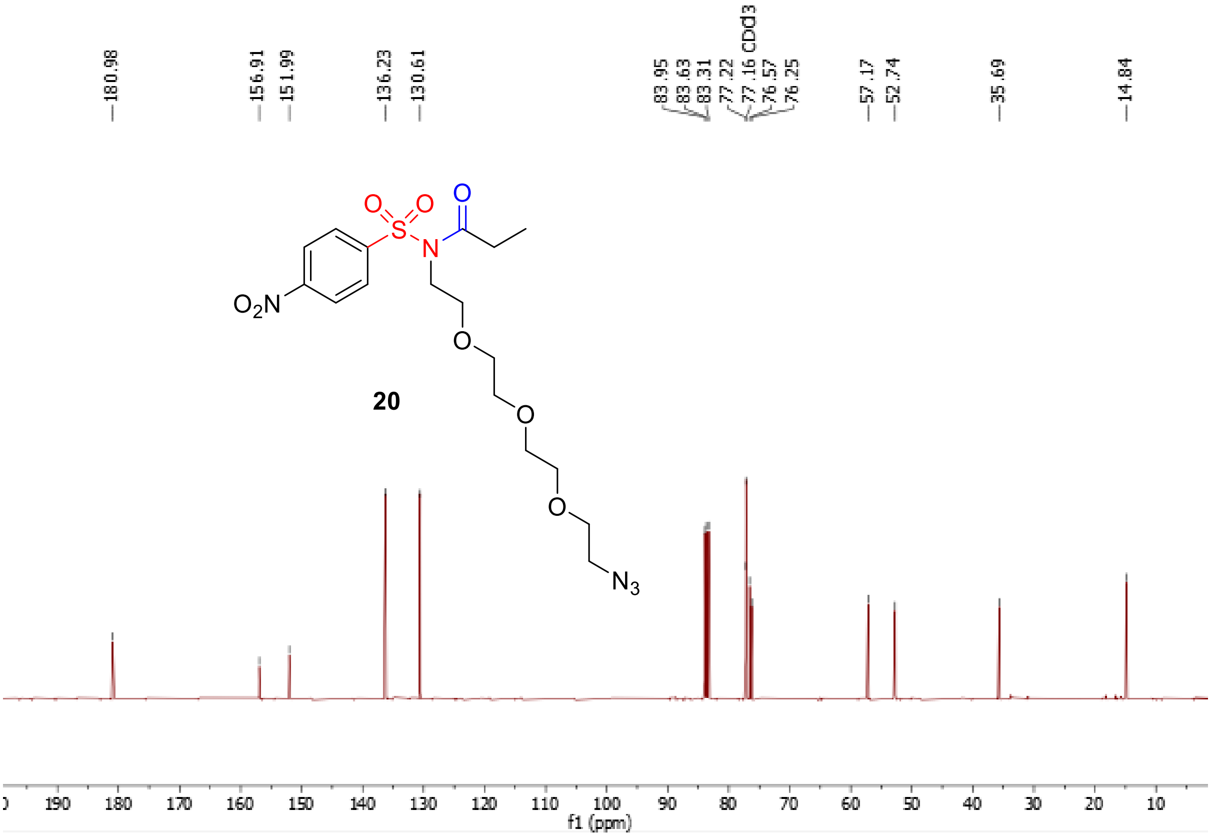

Supplement: OB-024-D6OB00339G-s001 [file OB-024-D6OB00339G-s001.pdf]
